# African ape carpal morphology is not too specialized to represent the ancestral hominin carpus

**DOI:** 10.1111/joa.70241

**Published:** 2026-09-17

**Authors:** Laura E. Hunter, Matthew W. Tocheri, Caley M. Orr, Biren A. Patel, Zeresenay Alemseged

**Affiliations:** ^1^ Department of Organismal Biology and Anatomy University of Chicago Chicago Illinois USA; ^2^ Department of Anthropology Lakehead University Thunder Bay Ontario Canada; ^3^ Human Origins Program National Museum of Natural History, Smithsonian Institution Washington DC USA; ^4^ Department of Cell and Developmental Biology University of Colorado School of Medicine Aurora Colorado USA; ^5^ Department of Anthropology University of Colorado Denver Denver Colorado USA; ^6^ Division of Integrative Anatomical Sciences, Department of Medical Education, Keck School of Medicine University of Southern California Los Angeles California USA; ^7^ Human and Evolutionary Biology Section, Department of Biological Sciences University of Southern California Los Angeles California USA

**Keywords:** carpals, human evolution, knuckle walking, locomotion, specialization

## Abstract

How and why hominins evolved key behaviors, such as obligate bipedalism and sophisticated tool production, are key questions in paleoanthropology. Crucial to this inquiry are accurate reconstructions of the morphology and behavior that characterized the panin–hominin last common ancestor (LCA). Because all extant hominines besides *Homo sapiens* knuckle‐walk, this rare hand posture has received special attention as a possible behavior in the LCA's locomotor repertoire. Arguments concerning knuckle‐walking ancestry in hominins typically examine functional morphology to explore possible adaptations to knuckle‐walking or leverage biological principles to evaluate the likelihood of certain evolutionary scenarios. Because wrist biomechanics in knuckle walking differ from those of other behaviors, carpals are frequently investigated for possible knuckle‐walking specializations. Here, we quantified carpal morphology using spherical harmonics analysis to reevaluate purported knuckle‐walking adaptations among an anthropoid sample with variable locomotor behaviors. We calculated Euclidean distances between behavioral pairs to investigate whether the carpals in knuckle‐walking taxa appear specialized relative to those of other taxa. Results show that nearly all hypothesized knuckle‐walking adaptations are broadly present in anthropoids, and several are specially shared with Asian apes. Some African ape carpal features are shared uniquely with humans and may signal knuckle‐walking ancestry. These results overall suggest that (1) the carpals of African apes are not specialized for knuckle walking; (2) to evolve knuckle walking, only few morphological changes to ancestral hominid carpals are necessary; and (3) an African ape‐like carpus is not too specialized to be a viable ancestral form from which the hominin carpus evolved.

## Graphical abstract

Knuckle walking is one hypothesized behavior of the Pan‐Homo last common ancestor. We analyzed carpal morphology, including Euclidean distances between behavioral pairs, to investigate whether the carpals of knuckle‐walking taxa appear relatively specialized. Results suggest that they are (1) not specialized for knuckle walking; (2) required few morphological changes to ancestral hominid carpals; and (3) are not too specialized to be a viable ancestral form from which the hominin carpus evolved.

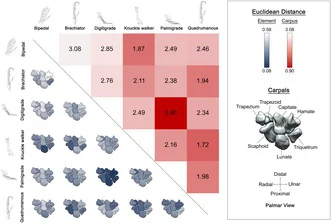

## 1 INTRODUCTION

Among extant primates, obligate terrestrial bipedalism and the sophisticated manufacture and use of tools are two features that characterize *Homo sapiens*. As such, the origins and evolution of these behaviors have long been central to paleoanthropological research (Broom & Robinson, 1949; Dart, 1953; Darwin, 1871). Yet, many issues pertaining to the patterns and timing of the evolution of these behaviors remain unresolved, including persistent debate over how the forelimbs were used by the panin–hominin last common ancestor (LCA), especially for locomotion (Almécija et al., 2021; Dainton & Macho, 1999; Kivell & Schmitt, 2009; Lovejoy, Simpson, et al., 2009; Richmond, 2006; Richmond & Strait, 2000; Tocheri et al., 2008). Putative habitual locomotor behaviors of the panin–hominin LCA and the earliest hominins include knuckle walking (Richmond & Strait, 2000), palmigrade quadrupedalism (Lovejoy, Simpson, et al., 2009), climbing and suspension (Stern, 1975), and arboreal bipedalism and suspension (Thorpe et al., 2007). Although there is increasing consensus that hominins evolved from an ancestor that likely included vertical climbing and/or suspensory behaviors in its locomotor repertoire (Green & Alemseged, 2012; Young et al., 2015; Prang et al., 2021; but see Lovejoy, Simpson, et al., 2009; White et al., 2009; Machnicki & Reno, 2020), it remains unclear whether other putative ancestral behaviors were also present.

Among the hypothesized behaviors in the locomotor repertoire of the panin–hominin LCA, knuckle walking has received special attention (e.g., Dainton & Macho, 1999; Goldstein & Sylvester, 2023; Lovejoy, Simpson, et al., 2009; Püschel et al., 2020; Richmond, 2006; Richmond et al., 2001; Sarmiento, 1988), likely because this behavior is (1) rare among mammals, (2) distinct from other terrestrial locomotor behaviors in primates (Orr, 2005), and (3) practiced habitually by both extant genera of African apes (*Pan* and *Gorilla*), the closest living relatives of *H. sapiens*. As a consequence, a search for knuckle‐walking traits in the skeletons of chimpanzees and gorillas has persisted for decades (Corruccini, 1978; Dainton & Macho, 1999; Daver et al., 2014; Goldstein & Sylvester, 2023; Jenkins & Fleagle, 1975; Lewis, 1973, 1985; Lovejoy, Simpson, et al., 2009; Marzke, 1983; McHenry, 1983; Püschel et al., 2020; Richmond, 2006; Richmond et al., 2001; Sarmiento, 1988; Schwartz & Yamada, 1998), with the aim of using these traits to ascertain whether knuckle walking played a role in the evolution of our lineage. Features of the forelimb musculature (e.g., Ito et al., 2024; Simpson et al., 2018; Tuttle, 1967, 1969) and long bones (e.g., Meyer et al., 2023; Orr, 2005; Richmond et al., 2001; Richmond & Strait, 2000) as well as the carpals (e.g., Corruccini, 1978; Dainton & Macho, 1999; Daver et al., 2014; Goldstein & Sylvester, 2023; Marzke, 1983; Püschel et al., 2020; Richmond, 2006; Sarmiento, 1988; Schwartz & Yamada, 1998) and metacarpals (e.g., Lovejoy, Simpson, et al., 2009; Sarringhaus et al., 2022; Simpson et al., 2018) have all been considered and leveraged to support claims that the earliest hominins did (Meyer et al., 2023; Richmond et al., 2001; Richmond & Strait, 2000) or did not (Kivell & Schmitt, 2009; Lovejoy, Simpson, et al., 2009; Lovejoy, Suwa, et al., 2009; White et al., 2009) knuckle walk and/or evolve from a knuckle‐walking ancestor.

Increasingly, arguments against or in support of a knuckle‐walking panin–hominin LCA have also been grounded by principles from paleontology, biomechanics, ontogeny, and evolutionary biology (Crompton et al., 2008; Doran, 1997; Kivell & Schmitt, 2009; Lovejoy, Suwa, et al., 2009; Richmond et al., 2001; Sarmiento, 1988; Simpson et al., 2018; Tuttle, 1967; White et al., 2009; Williams, 2010). The principle of parsimony is often invoked as a logical explanation in favor of the view that ape terrestriality and/or knuckle walking evolved only once in hominines (Richmond et al., 2001) and likewise in support of the idea that orthograde, extended‐knee bipedalism was less likely to evolve from pronograde, bent‐knee knuckle walking than from orthograde arboreal behaviors (Crompton et al., 2008). Researchers have relatedly discussed the role of contingency, phylogenetic inertia, and/or evolutionary history in shaping what morphology or behaviors were present or accessible throughout the evolution of the hominine clade (Richmond et al., 2001; Simpson et al., 2018). Ontogenetic and behavioral differences between *Pan* and *Gorilla* have also been used as evidence of independent origins of knuckle walking in these two genera (Doran, 1997; Kivell, 2007; Kivell & Schmitt, 2009; Sarmiento, 1988). Conversely, differences in patterns of morphological covariation between these two genera have been argued to make the case that knuckle‐walking traits cannot be easily assembled as a morphological suite of characters and therefore it is likely this behavior evolved only once in hominines (Williams, 2010).

In contrast to the search for knuckle‐walking traits shared between *Pan* and *Gorilla*, these studies are largely concerned with inferring what conditions could have led to the evolution of knuckle walking and how evidence gathered from extinct or extant apes may tip the scales in favor of one evolutionary scenario (e.g., a single knuckle‐walking origin) or another (e.g., convergent evolution of knuckle walking). In this class of arguments, the oldest and most enduring is the notion that the forelimbs of African apes are too specialized to serve as a reasonable hypothesized ancestral form of the hominin forelimb (Lovejoy, Simpson, et al., 2009; Lovejoy, Suwa, et al., 2009; Tuttle, 1967; White et al., 2015). However, few attempts have been made to formalize and quantify what specialized or “too specialized” specifically means in this context.

Although the carpus is only a part of the forelimb, its central role in forelimb posture biomechanics and thus in the debate concerning the evolution of knuckle walking makes it an ideal area to undertake this investigation. The main aims of this study are to (1) review the purported knuckle‐walking traits of the carpals and assess if they are truly distinctive in African apes and (2) interrogate the use of “specialized” within the field of paleoanthropology and reevaluate whether knuckle‐walking taxa appear to possess specialized wrist morphology, with the aim of further investigating the rest of the forelimb in future studies. We accomplish our aims by (1) using landmark‐free 3D methods to quantify and reevaluate the morphological features of the carpus often associated with knuckle walking, and (2) quantifying specialization as the distance in carpal morphology between locomotor groups and assessing how knuckle walking compares with other quadrupedal hand postures or locomotor behaviors, such as digitigrady and brachiation.

### 1.1 Knuckle‐walking features of the carpus

Within the forelimb, there are several osteological features which have been hypothesized as adaptations in response to the functional demands of knuckle walking. In the humerus, high humeral torsion in *Pan* and *Gorilla* is hypothesized to facilitate the parasagittal forelimb movement of knuckle walking (Arias‐Martorell et al., 2012; Larson, 1988; Richmond et al., 2001), and the site of attachment for the infraspinatus is expanded and more distally placed than in *Homo* and *Pongo*, hypothesized to stabilize the shoulder during knuckle walking (Arias‐Martorell et al., 2012; Green & Alemseged, 2012). Distal to the humerus, robust and ventrally curved ulnae have been associated with activation of wrist and elbow flexor muscles during knuckle walking (Meyer et al., 2023). In the hand, the metacarpal heads are dorsally expanded and possess dorsal ridges in knuckle‐walking taxa (Richmond et al., 2001; Sarringhaus et al., 2022; Susman, 1979), and variation in the relative dorsopalmar curvatures of the proximal and intermediate phalanges distinguishes knuckle‐walking taxa from those of other anthropoid primates in a manner reflective of differences in loading patterns between knuckle walkers and other taxa (Matarazzo, 2008). Within the forelimb, however, the wrist has received special attention in the search for knuckle‐walking traits, chiefly because its nature as a joint complex presents a challenge in interpreting functional morphology and because the wrist posture of knuckle walking is distinct among anthropoid primate behaviors.

**FIGURE 1:**
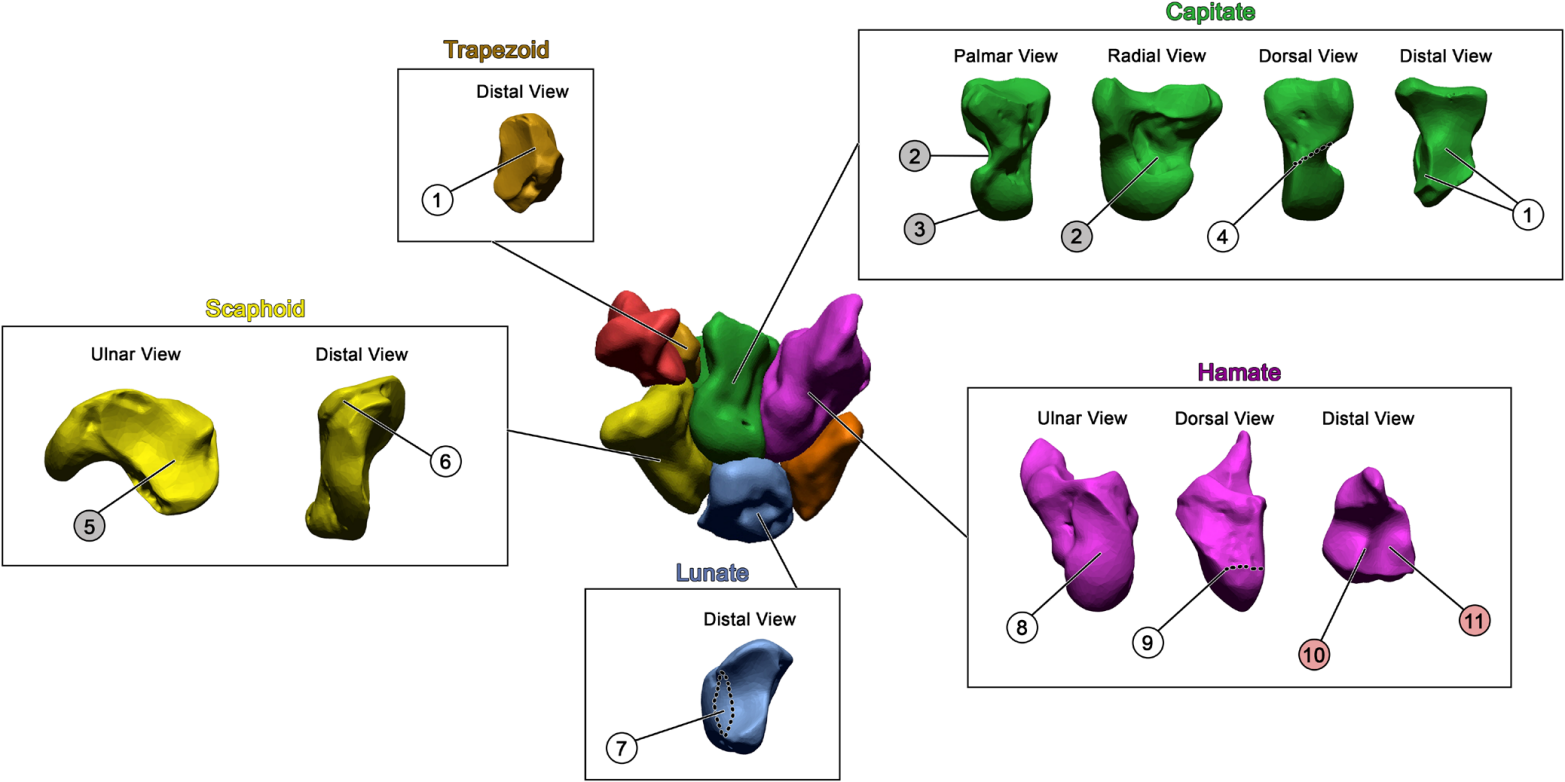
Features of carpal morphology hypothesized to be associated with knuckle walking, as demonstrated by a carpus of *Pan troglodytes* (KUPRI 461). Gray circles indicate features related to the “screw‐clamp” mechanism limiting midcarpal mobility. Pink circles indicate features related to proximodistal compressive loading. Features include: (1) complex metacarpal articular facets on the trapezoid and capitate with a more sagitally oriented facet for the second metacarpal on the capitate, (2) radioulnarly narrow capitate neck, (3) radially expanded capitate head, (4) proximally placed ridge on distal extent of the lunate facet on the capitate, (5) scaphoid–centrale fusion, (6) ridge at the boundary between the radius and trapeziotrapezoid facets on the scaphoid, (7) presence of a large articular facet for the hamate on the lunate, (8) concavoconvex, “spiral” articular facet for the triquetrum on the hamate, (9) proximally placed ridge on distal extent of the lunate facet on the hamate, (10) keel between the facets for the fourth and fifth metacarpals on the hamate, (11) larger fourth metacarpal facet on the hamate relative to the fifth metacarpal facet, (12) proximodistally short carpals (not pictured).

Specifically, knuckle walking differs from other proposed locomotor behaviors of the panin–hominin LCA in terms of the direction of mechanical loading imposed on the wrist. In contrast to the hyperextended wrist posture used during palmigrady, the knuckle‐walking hand posture holds the wrist in a slightly flexed, neutral, or slightly extended position, with only the dorsal aspect of the intermediate phalanges contacting the substrate (Finestone et al., 2018; Thompson, 2020; Tuttle, 1967). Because of this, knuckle walking likely requires muscular, ligamentous, and/or osteological features that limit further wrist extension (Figure 1). The role that the muscular and ligamentous anatomy of the wrist play in dictating the range of motion at these joints cannot be overstated (e.g., Ito et al., 2024; Potau et al., 2022; Selby et al., 2016; Simpson et al., 2018; Tuttle, 1967). However, these soft tissues do not preserve in fossils, and comparative studies across anthropoids remain scarce due to a dearth of available nonhuman primate cadaveric material, warranting a focus on osteological features which can be compared directly to fossil material. Because wrist extension can occur at the carpometacarpal, midcarpal, and/or radiocarpal joints, osteological features of the distal and proximal ends of the carpals along with the distal end of the radius have received the most attention in the search for extension‐limiting features of the wrist (Corruccini, 1978; Dainton & Macho, 1999; Kivell & Schmitt, 2009; Orr, 2005, 2017; Patel & Carlson, 2007; Püschel et al., 2020; Richmond, 2006; Richmond et al., 2001; Richmond & Strait, 2000; Sarringhaus et al., 2022; Tuttle, 1967).

In the carpometacarpal and radiocarpal joints, comparatively few osteological features have been hypothesized to play a functional role in knuckle walking. At the carpometacarpal joint, the articular facets for the second and third metacarpal joints are topographically complex and the facet for the second metacarpal on the capitate is sagitally oriented in *Pan* and *Gorilla*. This is in contrast to *H. sapiens* (Figure 1, Trait 1), in which these metacarpal facets on the trapezoid and capitate are planar and the second metacarpal facet on the capitate is oriented more distally (Clark, 1947; Lewis, 1973; Lovejoy, Simpson, et al., 2009; Marzke, 1983; Marzke & Marzke, 1987; McHenry, 1983; Orr, 2005; Tocheri et al., 2007). The topographic complexity of these facets in *Pan* and *Gorilla* has been cited as advantageous for resisting shear stresses, increasing wrist rigidity, and dissipating energy during knuckle walking (Lovejoy, Simpson, et al., 2009; Richmond et al., 2001). In the radiocarpal joint, a palmar tilt of the radius resulting in a dorsal ridge on its distal extent in *Pan* and *Gorilla* has been interpreted as functionally relevant to knuckle walking by acting as a bony buttress that halts movement in the radiocarpal joint during extension. Its presence in *Australopithecus* has been interpreted as evidence of knuckle walking not in this genus but in the panin–hominin LCA (Richmond et al., 2001; Richmond & Strait, 2000).

By contrast, the midcarpal joint has been studied extensively for knuckle walking traits (Lewis, 1972; McHenry, 1983; Lewis, 1985; Viegas et al., 1993; Marzke et al., 1994; Richmond, 2006; Kivell & Schmitt, 2009; Orr, 2017; Bird et al., 2024). Morphologically similar to the dorsal ridge on the distal radius, a ridge is also present at the border between the trapeziotrapezoid and radius facets of the scaphoid (Figure 1, Trait 6) in *Pan* and *Gorilla* (Tuttle, 1967; Lewis, 1972; Yalden, 1972; Lewis, 1985; Richmond & Strait, 2000; Richmond et al., 2001; Kivell & Schmitt, 2009). Some studies (Richmond, 2006; Tuttle, 1967) have interpreted this ridge as a bony buttress limiting the dorsal translation of the trapezoid and trapezium relative to the scaphoid, thus preventing extension of the radial side of the midcarpal joint. Similarly, there are proximally placed bony ridges on the dorsal aspects of the capitate and hamate where their articulations with the proximal carpal row end (Figure 1, Traits 4 & 9), limiting extension at the center and ulnar side of the midcarpal joint (Tuttle, 1967; Richmond et al., 2001; Orr, 2005; Richmond, 2006; Jenkins & Fleagle, 1975). In addition, contact between the hamate and lunate (Figure 1, Trait 7) has been variably (62–100%) observed in *Pan* and *Gorilla* (Marzke et al., 1994; Sarmiento, 1988) but is reduced or absent in palmigrade and digitigrade monkeys (Marzke et al., 1994; Schwartz & Yamada, 1998). Clinical research in *H. sapiens* found that it is associated with decreased midcarpal flexion–extension (Bain et al., 2015). Notably, however, this hamate–lunate articulation is common but variably present in *H. sapiens* (65%) and *Pongo* (87%) (Bain et al., 2015; Sarmiento, 1985; Viegas et al., 1993).

Most notable among the osteological features related to potentially limiting wrist extension in knuckle walkers are those associated with the “screw‐clamp mechanism,” a series of interactions among the scaphoid, lunate, and capitate that cause the proximal and distal carpal rows to interlock during extension (Conroy & Fleagle, 1972; Corruccini, 1978; Dainton & Macho, 1999; MacConaill, 1941; Orr, 2018). During the stance phase, the scaphoid acts as a stable surface against which the lunate becomes pinned through tightening of the triquetrolunate ligament, preventing any further movement. Simultaneously, the scaphoid supinates onto the head of the capitate and “locks” these bones into place, reducing articular contact between the capitate and the lunate (MacConaill, 1941; Tuttle, 1967; Lewis, 1973; Orr et al., 2010; Jenkins & Fleagle, 1975). Features identified as a part of this screw‐clamp mechanism in knuckle walkers include (1) reduced size of the capitate–lunate articular surfaces (Figure 1, Trait 7), providing evidence of limited articulation between these two elements (MacConaill, 1941; Tuttle, 1967; Lewis, 1973; Orr et al., 2010; Jenkins & Fleagle, 1975), (2) fusion of the scaphoid and centrale (Figure 1, Trait 5), thus forming a stable platform to pin the lunate (Marzke, 1971; Orr et al., 2010; Richmond et al., 2001; Tuttle, 1967), (3) a distinct, radioulnarly compressed capitate neck (Figure 1, Trait 2) between its proximal and distal ends (Clark, 1947; Kivell & Schmitt, 2009; Lewis, 1972, 1973; Tuttle, 1967), and (4) radial expansion of the capitate head (Lewis, 1985; Figure 1, Trait 3). The last two of these features contribute to form what is often referred to as capitate “waisting,” in which the proximodistal center of the capitate takes on a “cinched” appearance most evident in palmar view (Figure 1).

Features of the putative knuckle walking screw‐clamp mechanism are present not just in *Pan* and *Gorilla* but in early hominins as well. Capitate “waisting” appears evident in *Ardipithecus ramidus* (Lovejoy, Simpson, et al., 2009) and has been described in all *Australopithecus* species for which capitate morphology is known (Bush et al., 1982; Johanson, Taieb, & Coppens, 1982; Kivell, Churchill, et al., 2018; Lewis, 1973; McHenry, 1983), and prenatal or natal scaphoid–centrale fusion is dominant in all hominine taxa (Schultz, 1936; Napier, 1962; Marzke, 1971; Lewis, 1985; Sarmiento, 1988; Kivell & Begun, 2007; Tocheri et al., 2007; Larson et al., 2009; Jenkins & Fleagle, 1975; Kibii et al., 2011; Kivell et al., 2011, 2015; Kivell, Churchill, et al., 2018), with partial or no fusion occurring only rarely (Kivell, Rosas, et al., 2018; Sarmiento, 1994; Simpson et al., 2019). The presence of these features in fossil hominins indicates that these taxa possessed a screw‐clamp mechanism.

It should be noted, however, that trends in scaphoid–centrale fusion in primates suggest that this feature may be primarily phylogenetically rather than biomechanically driven (Kivell & Begun, 2007). Nonetheless, fusion between the scaphoid and centrale has also been proposed as an adaptation related to distributing forces through the carpus when it is compressively loaded during knuckle walking (Marzke, 1971; Püschel et al., 2020; Richmond et al., 2001; Tuttle, 1967). Unlike during suspensory behaviors, body weight compressively loads the wrist during knuckle walking (Carlson & Patel, 2006), specifically—in contrast to palmigrady—with all elements stacked roughly in‐line proximodistally. As such, other features of carpal morphology in addition to scaphoid–centrale fusion have been proposed as evidence of proximodistal compression in the wrist of knuckle‐walking taxa. One such feature is decreased carpal proximodistal lengths (Figure 1, Trait 12) relative to those of other taxa that include suspension in their locomotor repertoire (Almécija et al., 2015; Clark, 1947; Corruccini, 1977, 1978; Dainton & Macho, 1999; Daver et al., 2014; Goldstein & Sylvester, 2023; Kivell et al., 2013; Lewis, 1972; McHenry, 1983; Richmond et al., 2001; Sarmiento, 1988; Vanhoof et al., 2021). In fact, decreased proximodistal length of the ulnarly located carpals of *Gorilla* relative to those of *Pan* may reflect differences in knuckle‐walking frequency, biomechanics, or ontogenetic timing in these two genera (Almécija et al., 2015; Dainton & Macho, 1999; Vanhoof et al., 2021). Regardless, both knuckle‐walking genera display some features of the hamate that have been proposed as related to proximodistal compression during knuckle walking. These include (1) a larger fourth metacarpal facet relative to the fifth metacarpal facet (Figure 1, Trait 11), reflecting greater compression on the fourth digit during knuckle walking (Marzke et al., 1994; Tuttle, 1967), and (2) the presence of a keel between these two metacarpal facets (Figure 1, Trait 10), corresponding to the angle at which these digits are held relative to each other (Marzke, 1983; Richmond et al., 2001).

By investigating the carpal morphology of knuckle walkers in the context of anthropoid primate carpal morphological variation, the current study will provide a reassessment of these purported knuckle‐walking traits and may reveal hitherto undetected trends in their distribution across anthropoid taxa and hand posture/locomotor groups.

### 1.2 The bearing of specialization on the origin of knuckle walking in hominines

Although the hypothesis that the forelimbs of knuckle‐walking African apes are too specialized to adequately represent the ancestral hominin forelimb has endured for decades (Lovejoy, Simpson, et al., 2009; Tuttle, 1967; White et al., 2015) and the term is used frequently in the literature, paleoanthropologists rarely formalize their definition of “specialization.” As such, to test this hypothesis, a brief overview of how researchers in the field use this term is warranted.

In the context of primate locomotor behavior, the terms “specialized” and “specialization” are typically employed in interrelated four ways (Table 1). The first of these uses (**Use Type 1**) places “specialists” in opposition to “generalists,” referring to taxa as “specialized” when they are limited or restricted to a subset of possible niches or behaviors (i.e., “behavioral specialization”), often compared with a hypothesized ancestral condition. This use of the term “specialized” is aligned with its usage in the broader field of evolutionary biology and ecology (Colles et al., 2009; Holmes, 1976; Liow, 2007). It is difficult to ascertain whether it would be accurate to call knuckle walking specialized per **Use Type 1** or “behaviorally specialized.” There is biomechanical evidence suggesting that knuckle‐walking taxa possess a more limited ability to extend the wrist relative to other taxa (Tuttle, 1967, 1969; Sarmiento, 1988; Orr et al., 2010; Jenkins & Fleagle, 1975; Orr, 2017; Ito et al., 2024), which may be a particularly relevant limitation given the importance of wrist extension during manipulation, throwing, and tool production in humans (Hazelton et al., 1975; Volz et al., 1980; Williams et al., 2010). However, it is unclear how much of a limitation this imposes on the breadth of behaviors African apes are capable of relative to other nonhuman primates that may serve as candidates for representing the ancestral form of the hominin wrist. Where data are available, knuckle‐walking taxa appear to have an equal or greater diversity of habitual hand postures compared with most other anthropoid taxa and especially other extant hominoids, as they engage in terrestrial quadrupedalism as well as both above‐ and below‐branch arboreal locomotion (Almécija et al., 2021; Hunt, 2016; Thompson et al., 2018). Although not as adept in tool use and manufacture as *H. sapiens*, knuckle‐walking taxa are nonetheless capable tool users (Boesch & Boesch, 1990; Cebeiro & Key, 2023) and display dexterous manipulation during food processing (Byrne et al., 2001; Neufuss et al., 2019), indicating that any adaptations to knuckle walking they may possess do not significantly impair their capacity for manipulation relative to other nonhuman primates. In fact, knuckle‐walking taxa have been considered generalized relative to other extant hominoids in some studies (e.g., Harcourt‐Smith, 2013) and Almécija et al. (2021) even suggested that knuckle walking may have evolved to escape a “specialization trap.”

**TABLE 1:** Examples of the four common uses of “specialized” or “specialization” in published literature pertaining to hominoid locomotor behaviors. Emphasis added.

Use type	Definition	Example in literature
1	Limited or restricted to a subset of possible niches or behaviors, often compared with a hypothesized ancestral condition. “Behavioral”	“Early orangutans, on the other hand, became more and more **specialized** for activities in the forest canopy and eventually lost the potential to survive in habitats that are as diverse as those occupied by modern chimpanzees and gorillas” (Tuttle, 1967, p. 201)
2	Adaptation in response to selection for a certain behavior. “Anatomical”	“The hand of Ar. ramidus appears almost entirely primitive relative to the anatomical **specializations** seen in extant apes (for example, metacarpal elongation, elaboration of CJC articulations and ligaments…, etc.). *Ar. ramidus* establishes that these changes in the ape hand are independent **specializations** for arboreal access and terrestrial travel (vertical climbing, forelimb suspension, knuckle walking)” (Lovejoy, Simpson, et al., 2009, p. 70.e5)
3	Uncommon, either morphologically or based on behavioral observation. “Unusual”	“Most Miocene apes would have been able to use palmigrade postures, even if more **specialized** locomotor hand postures such as knuckle walking or digitigrade have been also [sic] proposed” (Daver et al., 2014, p. 127)
4	Distinct morphology, distant in morphospace from those of other taxa. “Relational”	“…limb **specialization**, which places apes and humans in relatively remote areas of morphospace” (Rolian, 2020, p. 702)

Other uses of the term “specialization” may have arisen from attempts to identify valuable proxies to infer the behavioral specialization of **Use Type 1**. For example, the term “specialization” is commonly used to refer to a morphological difference or “anatomical specialization” between one taxon or group and others (Table 1), particularly in cases where the morphological difference is hypothesized or inferred to be a derived adaptation in response to selection for a certain behavior (**Use Type 2**). In some sense, the term “specialization” in this case is related to specific behaviors and signals a departure from a more generalized condition but does not necessarily result in reducing the breadth of niches or behaviors possible (**Use Type 1**) for the organism with this “specialization.” One example of such a **Use Type 2** or “anatomical specialization” in primates is the highly modified dentition of the aye‐aye (*Daubentonia*); it is ostensibly a derived adaptation in response to selection for gouging trees to extract insect prey but does not limit the diet of aye‐ayes, which are broadly omnivorous and include both soft (e.g., fruits, nectar) and hard (e.g., nuts, seeds) foods in their diets (Sterling & McCreless, 2007). In the case of knuckle walking, the many putative knuckle‐walking adaptations discussed in the previous section would be “specializations” under this usage.

In discussing “anatomical specializations” of **Use Type 2**, there is an implicit assumption that the more common morphological condition represents the unspecialized or ancestral morphology. This assumption is shared with other uses of “specialized” and appears central to how many use the term. The notion that common features are ancestral or unspecialized makes intuitive sense; it is parsimonious and appears to have some basis in research that suggests specialized taxa are less evolvable (e.g., Rolian, 2020) or more vulnerable to extinction than other taxa (Simpson, 1984; Liow, 2007; but see Holmes, 1976; Colles et al., 2009) and therefore less likely to represent an ancestral form. Based on a similar assumption, “specialized” is sometimes used to refer to *behaviors* that are unusual, or **Use Type 3** (Table 1). Like **Use Type 2**, however, this use of the term appears neutral with regards to limiting behaviors. Because knuckle walking is an extremely uncommon behavior habitually practiced only by extant African apes and the giant anteater (Orr, 2005), it is undoubtedly specialized per **Use Type 3**. This usage, however, has little bearing on questions of evolvability or the relative likelihood of any ancestral form in particular.

A natural extension of “specialization” as functional adaptation (**Use Type 2** or “anatomical specialization”) is the use of “specialized” or “specialization” to refer to taxa with features that are morphologically distinct relative to those of other—usually closely related—taxa (**Use Type 4;** Table 1; Holmes, 1976; Liow, 2007; Rolian, 2020). The assumption implicit in **Use Type 4** is that specialized taxa have either undergone very extreme changes in a few morphological features (e.g., finch beak length and curvature) or accumulated enough subtler anatomical specializations (**Use Type 2**) to make them morphological outliers in the context of their clade (i.e., “relational specialization”). In such circumstances, it is possible that their morphological distance from other taxa has some effect on the breadth of behaviors or niches available to them. This use of morphological distinctness to determine or define specialization is common in paleobiology or in endangered or cryptic clades because sufficient niche/behavioral data to directly assess ecological or behavioral specialization (**Use Type 1**) in these groups is lacking (Liow, 2007; Rolian, 2020; Simpson, 1984). In addition to ecological or behavioral specialization (**Use Type 1**) studies, research following the relational specialization **Use Type 4** appear to form the basis for the idea of a “specialization trap”; the ability of these specialized taxa to shift toward new adaptive peaks is hindered by an accumulation of specializations that pushed them far from a generalized, evolvable form (Rolian, 2020).

When researchers state that the forelimbs of knuckle walkers are “too” specialized to represent the ancestral hominin form (Lovejoy, Suwa, et al., 2009; Tuttle, 1967; White et al., 2009), it appears that they are referring to a preponderance of anatomical specializations (**Use Type** 2) or relational specialization (**Use Type 4**). This implies that morphological distinctness is hindering evolvability or that the morphological distance that must be overcome to evolve the derived morphology is too great. By quantifying the morphological distance among different locomotor groups of anthropoid primates, the current study assesses the degree to which the wrist of the knuckle‐walking African apes is truly specialized compared with those of other primates.

## 2 METHODS

### 2.1 Sample

The sample (Table 2) consists of the scaphoid–centrale, lunate, triquetrum, trapezium, trapezoid, capitate, and hamate (*n* = 2037 bones) from 291 anthropoid individuals representing the following locomotor behaviors and/or hand posture types (Table 2): knuckle walking, bipedalism, brachiation, quadrumanous/slow climbing, and palmigrade and digitigrade pronograde quadrupedalism (Patel, 2010a; Patel & Carlson, 2007). It should be noted that some of these behaviors and/or hand posture types are represented by a single taxon (bipeds are *H. sapiens*) or clade (e.g., quadrumanous/slow climbing taxa are all orangutans), but these taxa will be referred to chiefly by their locomotor group designation throughout. The specimens come from the skeletal collections of the following institutions: AMNH, American Museum of Natural History (New York, NY, USA); ANSP, Academy of Natural Sciences of Drexel University (Philadelphia, PA, USA); LACM, Natural History Museum of Los Angeles County (Los Angeles, CA, USA); MCZ, Harvard University Museum of Comparative Zoology (Cambridge, MA, USA); RMCA, Royal Museum for Central Africa (Tervuren, Belgium); SBU, Department of Anatomical Sciences of Stony Brook University (Stony Brook, NY, USA); USNM, Smithsonian Institution's National Museum of Natural History (Washington, DC, USA); and KUPRI, Kyoto University's Primate Research Institute (Kyoto, Japan). Effort was made to sex balance each species, but this was not always possible. Significant sex bias was present for *Gorilla beringei* (~83% male) and *Pongo abelii* (~78% female). All specimens were adults (defined by eruption of the third molar per (Kivell, 2007) or by epiphyseal fusion of the long bones) with carpals that were fully ossified and showed no clear signs of pathology. Most specimens were wild, but some captive (*n* = 18) and unknown‐locality (*n* = 27) individuals were included to bolster the sample size of certain taxa. Because the scaphoid and centrale are separate bones in all sampled taxa except for hominines (*Homo, Pan, Gorilla*), these bones in non‐hominine taxa were digitally fused to make these structures homologous for comparative analysis (Figure S1). This was done using the Align Between Scan Data and Combine tools in Geomagic Design X v. 2020 software. In cases where these elements were unfused but remained articulated via ligaments, they were scanned and segmented as a unit.

**TABLE 2:** Comparative sample with number of individuals, sex and captivity distributions, mean body mass, and observed locomotor behavior.

Species	# individuals	Sex*	Captivity status**	Mean body mass (kg)^†^	Locomotion/hand posture^††^
*Homo sapiens*	50	14/12/24	NA	54.4–62.2	Bipedal
*Pan paniscus*	12	4/8/0	0/12/0	33.2–45.0	Knuckle walking
*Pan troglodytes*	13	6/7/0	0/13/0	40.37–49.57	Knuckle walking
*Gorilla gorilla*	14	7/5/2	0/14/0	71.5–170.4	Knuckle walking
*Gorilla beringei*	11	10/1/0	0/11/0	97.5–162.5	Knuckle walking
*Pongo pygmaeus*	40	17/20/3	12/25/3	35.8–78.5	Quadrumanous
*Pongo abelii*	9	2/7/0	0/9/0	35.6–77.9	Quadrumanous
*Symphalangus syndactylus*	11	4/7/0	1/6/4	10.7–11.9	Brachiation
*Hoolock hoolock*	11	3/7/1	2/8/1	6.88–6.87	Brachiation
*Hylobates agilis*	4	1/2/1	0/3/1	5.82–5.88	Brachiation
*Hylobates lar*	17	8/8/1	0/14/3	5.34–5.9	Brachiation
*Cercopithecus mitis*	11	4/6/1	0/10/1	4.25–7.93	Palmigrade
*Macaca fascicularis*	13	5/7/1	0/11/2	3.59–5.36	Palmigrade
*Nasalis larvatus*	13	6/4/3	0/12/1	9.82–20.4	Palmigrade
*Sapajus apella*	12	6/4/2	0/12/0	2.52–3.65	Palmigrade
*Macaca mulatta*	13	7/6/0	0/10/3	7.09–9.36	Digitigrade
*Papio anubis*	13	7/4/2	0/12/1	13.3–25.1	Digitigrade
*Papio hamadryas*	12	6/1/5	3/3/6	10.65–18.95	Digitigrade
*Erythrocebus patas*	12	7/5/0	0/12/0	6.5–12.4	Digitigrade
Total # Individuals	291				
Total # Specimens	2037				

* Male/female/unknown.

** Captive/wild/unknown.

^†^ Smith and Jungers (1997).

^††^ Fleagle (1998); Patel (2010a).

### 2.2 3D data collection procedures

A large sample of carpal 3D digital meshes was reconstructed from μCT and 3D structured light surface scans. μCT scanning was conducted with a Pheonix V | Tome | X S scanner at voltage = 100 kV, current = 180 μA, exposure timing = 150 ms. Scan voxel size ranged from ~30 to ~60 μm. Structured light scanning was conducted with the Creaform Go! Scan 3D hand scanner or Artec3D High Precision Desktop 3D scanner. Additional carpal surface models were generated from CT, μCT, or laser surface scans, with 3D model construction protocols detailed elsewhere (Fernández et al., 2015; Patel et al., 2017; Wennemann et al., 2022).

### 2.3 Morphological analysis

**FIGURE 2:**
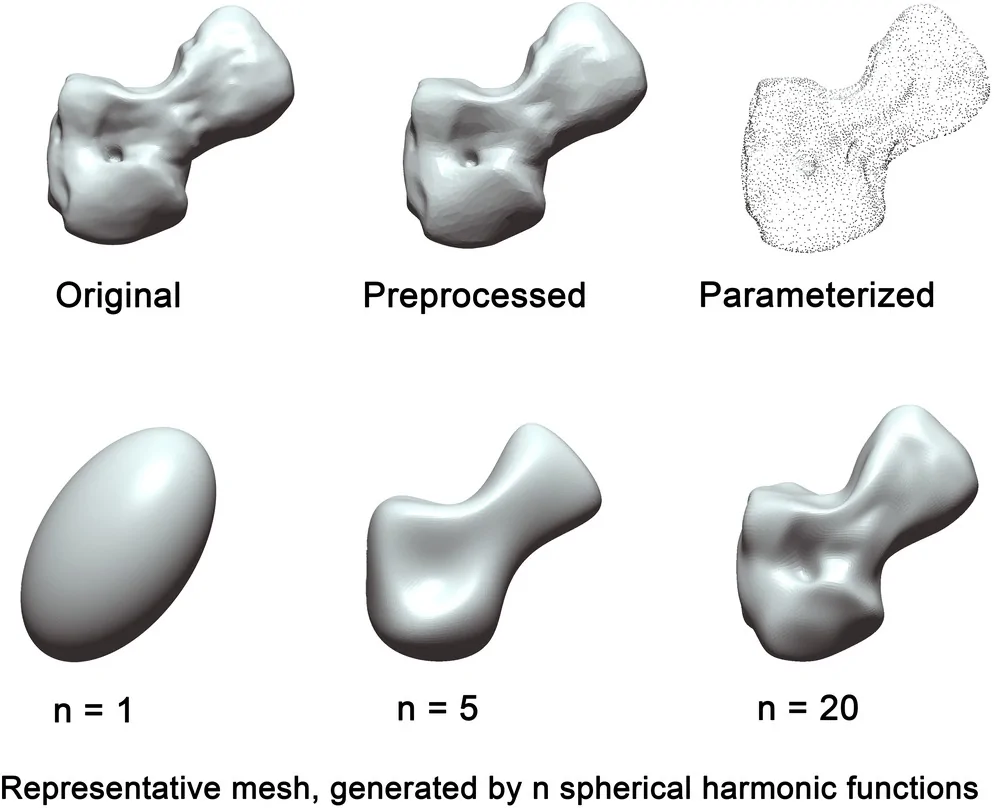
Demonstration of SPHARM method and morphological accuracy. Top, left to right: Original scan of a *Gorilla beringei* specimen (USNM 176225); same scan preprocessed to a manifold, 10 k poly‐face mesh; and the parameterized mesh with all vertices placed in coordinate space, visualized here as a point cloud. Bottom: Reconstructed scan using 1, 5, and 20 spherical harmonic functions.

Carpal 3D whole‐bone morphology was evaluated using a spherical harmonics (SPHARM) approach (Figure 2). Such a whole‐bone approach has been successfully used previously to investigate the morphology of primate tarsals (Harper et al., 2022; Harper et al., 2023; Llera Martín et al., 2022) and manual phalanges (Goldstein et al., 2026). SPHARM quantifies the morphological characteristics of a 3D digital mesh by first establishing spherical shape–space coordinates for each vertex of the mesh to parameterize its overall appearance. The clustering and location of vertices on a mesh within this coordinate system reflect the specific shape properties of the mesh, now standardized to the SPHARM coordinate system. The coordinates for the digital mesh that SPHARM establishes (i.e., a “map” of overall mesh morphology) can be conceptually thought of as a landmark system in which each vertex of the digital mesh is a “landmark” whose coordinate location has been identified in a spherical/polar shape space. Once SPHARM has parameterized the mesh in this way, it reconstructs the appearance of the mesh using a series of spherical harmonic functions. These spherical harmonic functions hierarchically describe the global and local shape properties of the mesh, with the value of their coefficients dictating the details of overall morphology. SPHARM can reconstruct a mesh using any number of spherical harmonic functions, with increased accuracy as the number increases. A digital mesh represented by a single spherical harmonic function will resemble an ellipsoid with the same approximate dimensions of the original mesh, whereas the same mesh represented by 20 spherical harmonic functions will closely but not perfectly resemble the original mesh (Figure 2). Because spherical harmonic functions are standardized with only their coefficients changing in value to replicate the morphology of the original mesh, a vector of their coefficients can be used to quantify the morphology of the mesh and vectors from multiple meshes can be compared with assess similarities and differences among meshes in overall morphology. These mesh comparisons are contingent on identical orientation, which can be accomplished via the placement of five homologous landmarks across each mesh before SPHARM processing, followed by a subsequent step during SPHARM processing which further minimizes the root mean squared distance between corresponding parts of the surfaces of all meshes. SPHARM can also be described as a 3D extension of two‐dimensional Elliptical Fourier analysis (Kuhl & Giardina, 1982), with specifically spherical harmonic functions as its basis functions.

Because SPHARM analyzes each mesh in its entirety, this method is more sensitive to differences in mesh characteristics across a sample than traditional geometric morphometric methods that rely on landmarking. Thus, all digitized meshes of sampled elements must possess approximately the same number of faces, must be watertight and manifold, and must not have any holes, folded or dangling faces, or internal geometry (Brechbühler et al., 1995; Shen et al., 2009). To ensure data compatibility with SPHARM, all digitized meshes underwent a processing pipeline in Geomagic Design X 2020. This included removing internal geometry and making each mesh watertight using the Fill Holes and Global Remesh tools, reducing each mesh to approximately 10,000 faces using the Decimate tool, and “healing” any abnormal faces using the Healing Wizard tool. Once this process was complete, all meshes were landmarked in MeshLab to standardize their orientation so that SPHARM would characterize morphology “homologously” across the sample. For each carpal, five landmarks were applied at locations intentionally dispersed across the entire element to provide the most informative possible data for orientation. All specimens were landmarked by L.E.H; intra‐observer error for each carpal was assessed by repeating landmark placement 10 times on one *Homo sapiens* individual. Across all carpals, the mean distance between identical landmarks placed across the error sample was 0.27 mm, with a maximum of 1.81 mm. Lists of these landmarks are outlined in Figures S3–S9. These landmarks were applied exclusively for mesh orientation and were not incorporated into the SPHARM analysis.

**FIGURE 3:**
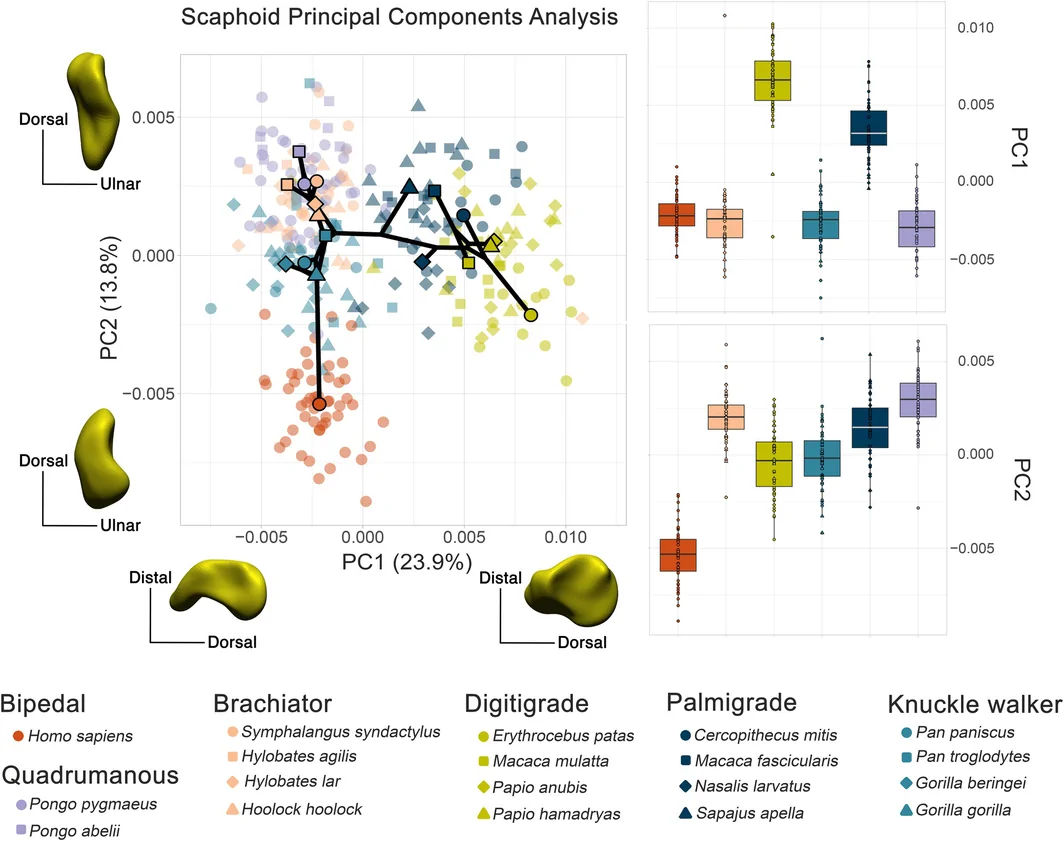
PCA phylomorphospace/scatterplot and box‐and‐whisker plots of PCs 1 and 2 for the scaphoid. PC1 morphs are in ulnar view with distal up. Lower values of PC1 are associated with proximodistally short, dorsopalmarly deep scaphoids with long tubercles, whereas higher values of PC1 are associated with proximodistally long, dorsopalmarly deep scaphoids with short tubercles. PC2 morphs are in distal view with dorsal up. Lower values of PC2 are associated with open capitates facets (i.e., a concave and “reduced” distal edge) and absence of a ridge between the radius and trapeziotrapezoid facets, whereas higher values of PC2 are associated with a closed capitate facet and presence of a ridge between the radius and trapeziotrapezoid facets. Articulated *Gorilla gorilla* (KUPRI 23) partial hand (DP1‐5, IP 2–5, and pisiform absent) included to display location of scaphoid within the carpus. Vertices of the phylomorphospace correspond with species means. Boxes represent first and third quartiles, whiskers extend 1.5× the interquartile range, horizontal lines represent the median of the sample distribution, and symbols represent individual data points.

**FIGURE 4:**
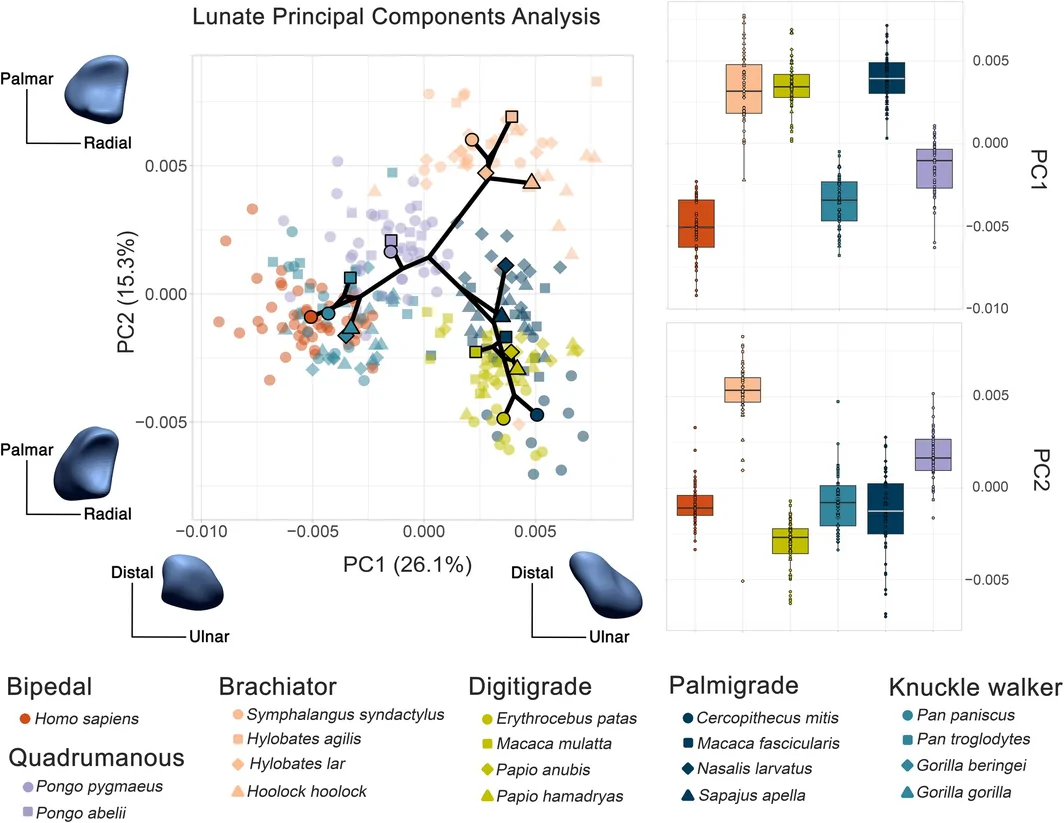
PCA phylomorphospace/scatterplot and box‐and‐whisker plots of PCs 1 and 2 for the lunate. PC1 morphs are in palmar view with distal up. Lower values of PC1 are associated with radioulnarly wide lunates with capitate facets in‐line with the lunate body, whereas higher values of PC1 are associated with radioulnarly narrow lunates with capitate facets that appear radially displaced relative to the lunate body. Lower values of PC2 morphs are in distal view with palmar up. PC2 are associated with defined scaphoid facets and radioulnarly wide, proximoulnarly listing distal facets with more hamate than capitate articulation. whereas higher values of PC2 are associated with a lack of scaphoid facet definition and radioulnarly narrow distal facets with no hamatolunate contact. Articulated *Gorilla gorilla* (KUPRI 23) partial hand (DP1‐5, IP 2–5, and pisiform absent) included to display location of lunate within the carpus. Vertices of the phylomorphospace correspond with species means. Boxes represent first and third quartiles, whiskers extend 1.5× the interquartile range, horizontal lines represent the median of the sample distribution, and symbols represent individual data points.

**FIGURE 5:**
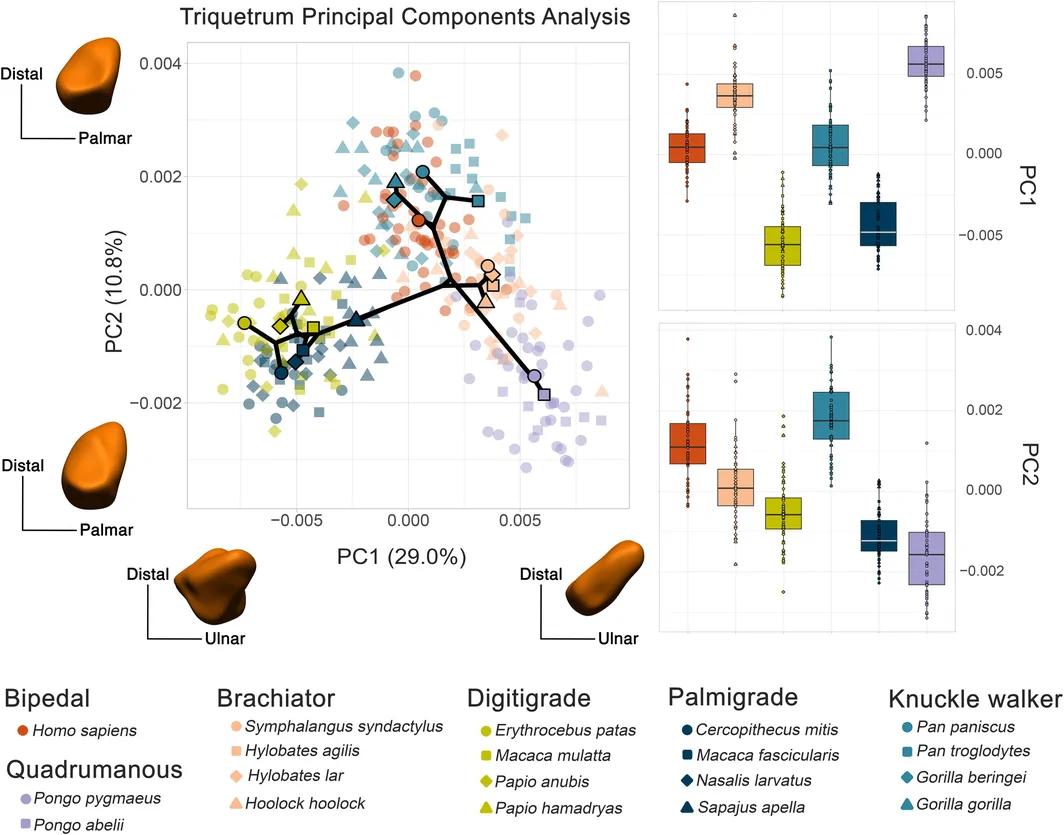
PCA phylomorphospace/scatterplot and box‐and‐whisker plots of PCs 1 and 2 for the triquetrum. PC1 is in palmar view with distal up. Lower values of PC1 are associated with dorsopalmarly deep triquetra with ulnar articulation and large, proximally oriented pisiform facets, whereas higher values of PC1 are associated with proximodistally long triquetra with small, palmarly oriented pisiform facets. PC2 is in radial view with distal up. Lower values of PC2 are associated with a pointed, nonarticular ulnodistal extension and convex lunate facets, whereas higher values of PC2 are associated with a lack of nonarticular ulnodistal protuberance and concave lunate facets. Articulated *Gorilla gorilla* (KUPRI 23) partial hand (DP1‐5, IP 2–5, and pisiform absent) included to display location of triquetrum within the carpus. Vertices of the phylomorphospace correspond with species means. Boxes represent first and third quartiles, whiskers extend 1.5× the interquartile range, horizontal lines represent the median of the sample distribution, and symbols represent individual data points.

**FIGURE 6:**
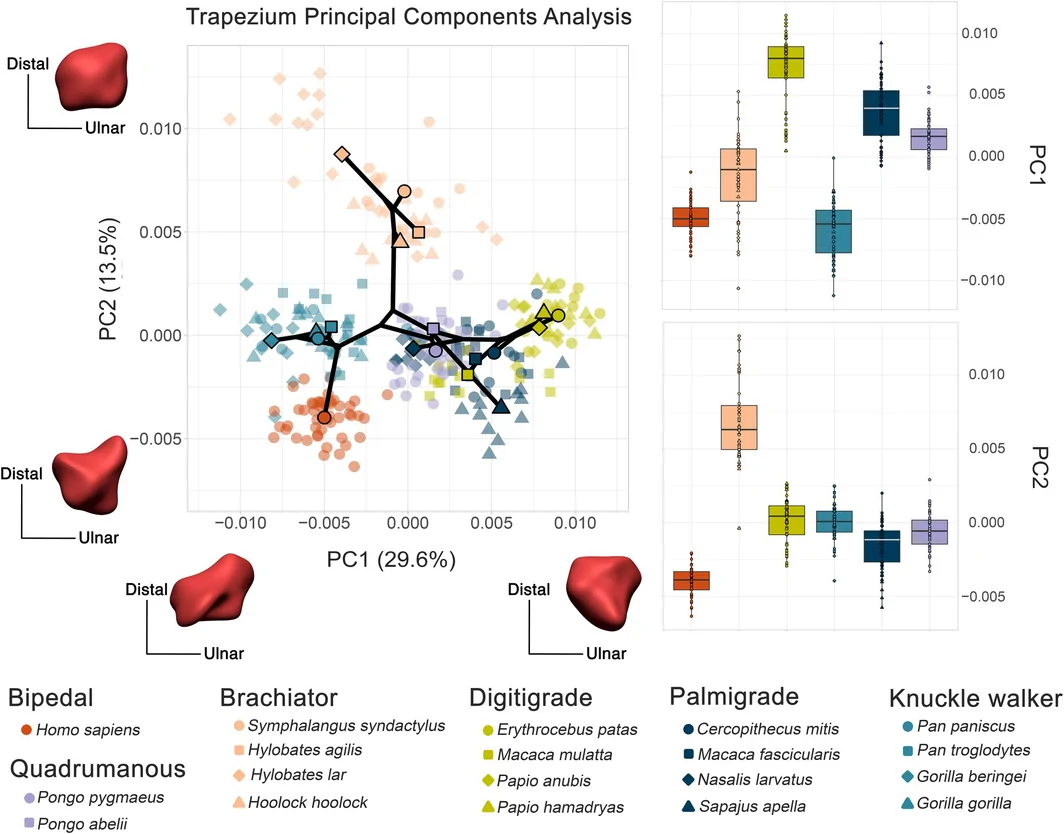
PCA phylomorphospace/scatterplot and box‐and‐whisker plots of PCs 1 and 2 for the trapezium. PC1 is in palmar view with distal up. Lower values of PC1 are associated with proximodistally short, dorsopalmarly deep trapezia with large tubercles, whereas higher values of PC1 are associated with proximodistally long, dorsopalmarly shallow trapezia with small tubercles. PC2 is in palmar view with distal up. Lower values of PC2 are associated with large, palmarly expanded, saddle‐shaped first metacarpal, whereas higher values of PC2 are associated with radially convex first metacarpal facets. Articulated *Gorilla gorilla* (KUPRI 23) partial hand (DP1‐5, IP 2–5, and pisiform absent) included to display location of trapezium within the carpus. Vertices of the phylomorphospace correspond with species means. Boxes represent first and third quartiles, whiskers extend 1.5× the interquartile range, horizontal lines represent the median of the sample distribution, and symbols represent individual data points.

**FIGURE 7:**
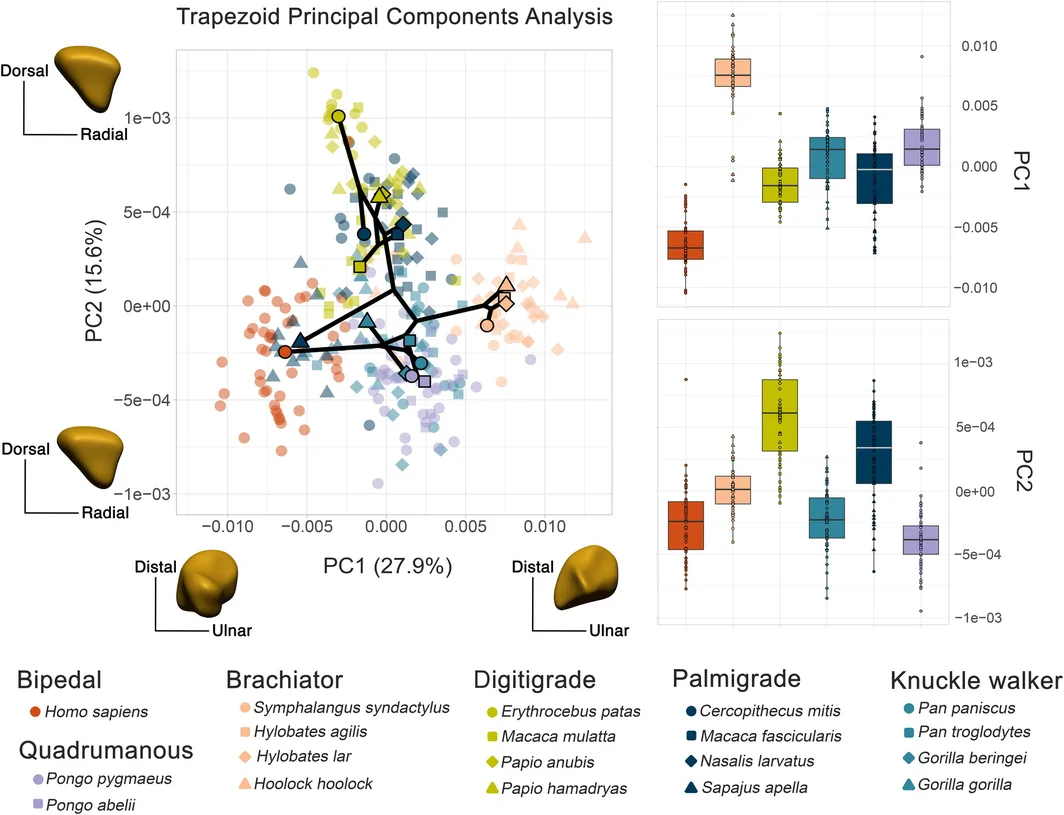
PCA phylomorphospace/scatterplot and box‐and‐whisker plots of PCs 1 and 2 for the trapezoid. PC1 is in palmar view with distal up. Lower values of PC1 are associated with dorsopalmarly deep trapezoids with more planar, distally oriented second metacarpal facets and radioulnarly broad palmar beaks, whereas higher values of PC1 are associated with dorsopalmarly shallow trapezoids with more mediolaterally oriented second metacarpal facets and extremely tapered palmar beaks resulting from acutely angled scaphoid and trapezium facets. PC2 is in proximal view with dorsal up. Lower values of PC2 are associated with a scaphoid facet with a palmar extent that lists more palmoradially, whereas higher values of PC2 are associated with a scaphoid facet with a palmar extent that does not list palmoradially. Articulated *Gorilla gorilla* (KUPRI 23) partial hand (DP1‐5, IP 2–5, and pisiform absent) included to display location of trapezoid within the carpus. Vertices of the phylomorphospace correspond with species means. Boxes represent first and third quartiles, whiskers extend 1.5× the interquartile range, horizontal lines represent the median of the sample distribution, and symbols represent individual data points.

**FIGURE 8:**
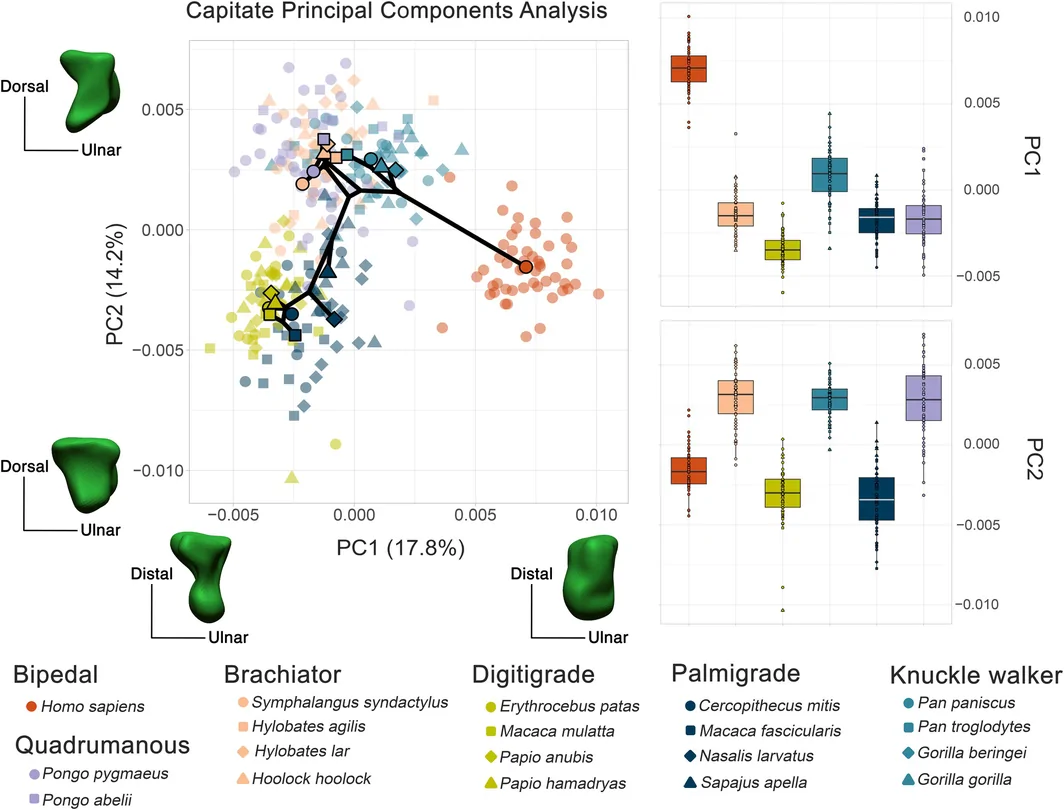
PCA phylomorphospace/scatterplot and box‐and‐whisker plots of PCs 1 and 2 for the capitate. PC1 is in palmar view with distal up. Lower values of PC1 are associated with radioulnarly wide capitate heads and necks, whereas higher values of PC1 are associated with radioulnarly narrow, “waisted” necks and heads. PC2 is in distal view with dorsal up. Lower values of PC2 are associated with dorsopalmarly flat but complex third metacarpal facets and palmarly restricted second metacarpal facets, whereas higher values of PC2 are associated with dorsopalmarly “cupped” but simple third metacarpal facets and second metacarpal facets spanning the entire dorsopalmar length of the bone. Articulated *Gorilla gorilla* (KUPRI 23) partial hand (DP1‐5, IP 2–5, and pisiform absent) included to display location of capitate within the carpus. Vertices of the phylomorphospace correspond with species means. Boxes represent first and third quartiles, whiskers extend 1.5× the interquartile range, horizontal lines represent the median of the sample distribution, and symbols represent individual data points.

**FIGURE 9:**
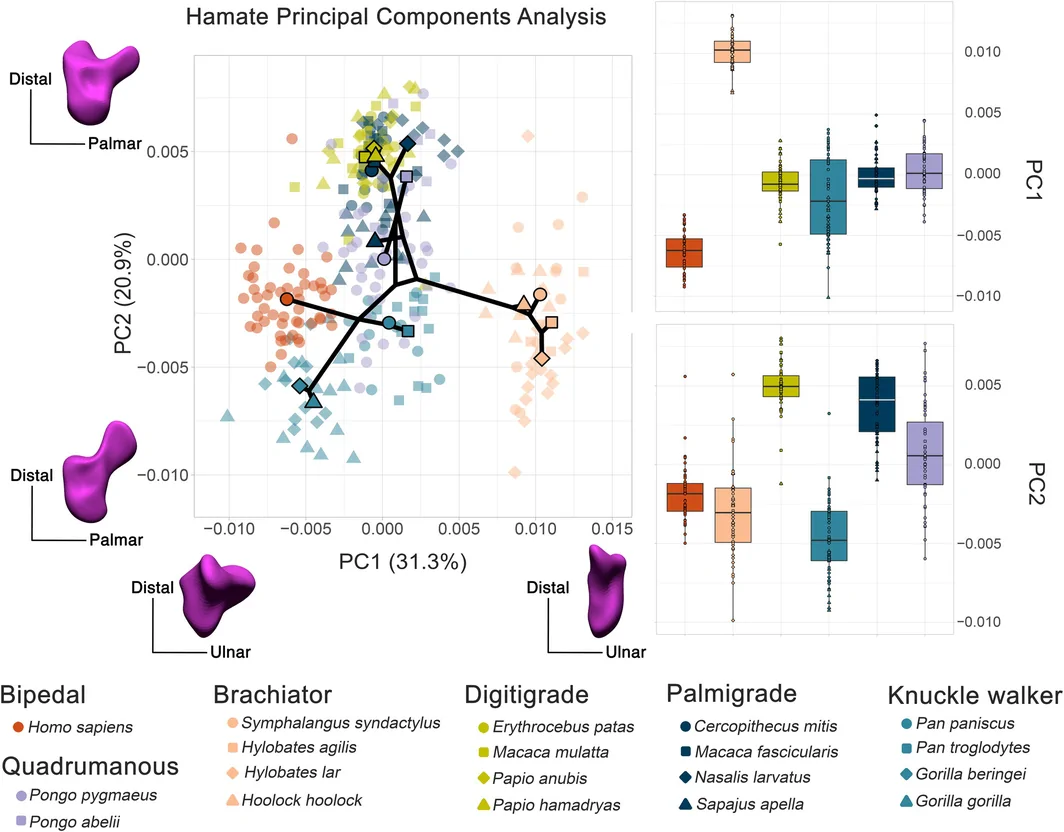
PCA phylomorphospace/scatterplot and box‐and‐whisker plots of PCs 1 and 2 for the hamate. PC1 is in palmar view with distal up. Lower values of PC1 are associated with proximodistally long hamates with distally oriented hamuli and entirely convex triquetral facets, whereas higher values of PC1 are associated with radioulnarly wide hamates with palmarly oriented hamuli and concavoconvex triquetral facets. PC2 is in radial view with distal up. Lower values of PC2 are associated with large hamuli and dorsopalmarly flat metacarpal facets, whereas higher values of PC2 are associated with small hamuli and highly dorsopalmarly concave metacarpal facets. Articulated *Gorilla gorilla* (KUPRI 23) partial hand (DP1‐5, IP 2–5, and pisiform absent) included to display location of hamate within the carpus. Vertices of the phylomorphospace correspond with species means. Boxes represent first and third quartiles, whiskers extend 1.5× the interquartile range, horizontal lines represent the median of the sample distribution, and symbols represent individual data points.

SPHARM analysis was conducted in MATLAB v.2022b software following (Shen et al., 2009). A set of 20 spherical harmonic functions was chosen to represent each digital mesh, based on visual assessment of how well these representations approximate the morphology of the original element (Figure 2). Principal components (PC) analysis was used to summarize the results of SPHARM and explore variation in carpal anatomy. Polygonal mesh warps were constructed in MATLAB to visualize extreme PC values (Figures 3, 4, 5, 6, 7, 8, 9 and S9–S15). The first 20 PCs were selected for investigation and subsequent analysis because 20 PCs explained ~85% of the total variance for each element (83.8–88.1%) and all subsequent PCs (e.g., PC21, PC22…) each explained <0.5% of total variance. Significant differences in the scores of the first 20 PCs among each extant species and locomotor/hand posture group were analyzed via Wilcox tests with Bonferroni correction for each individual PC and Hotelling *t*‐test for PCs 1–20 (Tables S1–S7). PCs which explain the most variance (i.e., PC1 and PC2) were selected for discussion.

Following SPHARM analysis, the dataset was closely examined anatomically to (1) validate morphological trends detected via SPHARM analysis and (2) identify any morphological trends in traits of interest which may not have been detected and quantified by SPHARM analysis. Although it was not possible to study the original material for all specimens in the sample, this close anatomical examination included physical inspection of several of the original bones and visual inspection of all digitized meshes included in the study.

To calculate morphological distinctiveness between hand posture groups, Euclidean distances were measured between group averages. Average values of PCs 1–20 were calculated for each locomotor group and were Z‐scored and weighted based on % variance. This was done to standardize the morphospace centroid sizes across all carpals so that each bone is weighted equally in the full carpus analyses. This method of rescaling the centroid size of the PC morphospace does not impact the relative positions of individual points. With these values that capture the average morphology for each locomotor group, the Euclidean distances between each pair of locomotor groups were calculated for each bone (e.g., the Euclidean distance between the average capitate morphology of bipedal and brachiating taxa) and for the full carpus (e.g., the Euclidean distance between the average carpus of digitigrade and quadrumanous taxa). Furthermore, the mean and variance in Euclidean distance among all locomotor behavior/hand posture groups for each bone was calculated to quantify the extent to which each element varies in morphology relative to locomotor behavior. These Euclidean distance metrics provide a simple scalar value that quantifies the morphological “distinctness” of each locomotor/hand posture group based on approximately 85% of the total variance in the sample, approximating a percent variance cutoff commonly employed in geometric morphometric studies (Zelditch et al., 2012).

## 3 RESULTS

### 3.1 SPHARM principal components analysis

#### 3.1.1 Scaphoid

The first 20 PCs explain 85.3% of variance in the scaphoid sample, PCs 1–4 explain 54.0%, and PCs 1 and 2 explain 37.7%.

PC1 (23.9% of variance) reflects (1) proximodistal and dorsopalmar dimensions and (2) tubercle dorsopalmar length, with bipedal, knuckle walking, quadrumanous, and brachiator groups possessing proximodistally short, dorsopalmarly deep scaphoids with long tubercles whereas non‐hominoids, especially digitigrade non‐hominoids, possess proximodistally long, dorsopalmarly deep scaphoids with short tubercles (Figures 3 and S9). PC1 distinguishes (1) digitigrade taxa from all other locomotor behaviors (all *p* < 0.0001) and (2) palmigrade taxa from all other locomotor behaviors (all *p* < 0.0001). PC1 fails to distinguish between bipedal, brachiating, knuckle walking, and quadrumanous taxa (*p* ≥ 0.35; Table S1).

PC2 (13.8%) reflects (1) capitate facet “openness” (i.e., a concave and “reduced” distal edge) and (2) ridge size where the trapeziotrapezoid and radius facets meet. Bipeds possess open facets and no ridge, contrasting with other groups, which possess closed facets and ridges where the trapeziotrapezoid and radius facets meet (Figures 3 and S9). PC2 distinguishes bipeds from all other locomotor behaviors (all *p* < 0.0001). PC2 fails to distinguish between digitigrade and knuckle‐walking taxa (*p* = 1) and between brachiating and quadrumanous or palmigrade taxa (*p* ≥ 0.38), although it distinguishes between palmigrade and quadrumanous taxa (*p* < 0.0001; Table S1).

#### 3.1.2 Lunate

The first 20 PCs explain 85.1% of variance in the lunate sample, PCs 1–4 explain 56.0%, and PCs 1 and 2 explain 41.3%.

PC1 (26.1% of variance) reflects (1) radioulnar width and (2) radioulnar location of the capitate facet relative to the lunate body, with bipedal, knuckle walking, quadrumanous, and brachiator groups possessing radioulnarly wide lunates with capitate facets in‐line with the lunate body whereas palmigrade and digitigrade groups possess radioulnarly narrow lunates with capitate facets that appear radially displaced relative to the lunate body (Figures 4 and S10). PC1 distinguishes (1) bipeds from all other locomotor behaviors (all *p* < 0.0001), (2) knuckle walkers from all other locomotor behaviors (all *p* < 0.0001), and (3) quadrumanous specimens from those of all other locomotor behaviors (all *p* < 0.0001). PC1 fails to distinguish between brachiating, palmigrade, and digitigrade groups (all *p* = 1; Table S2).

PC2 (15.3%) reflects (1) scaphoid facet definition and (2) the shape of the distal facet, including degree of hamatolunate contact, with brachiators possessing defined scaphoid facets and radioulnarly wide, proximoulnarly listing distal facets with more hamate than capitate articulation. At the other extreme, several palmigrade and digitigrade specimens lack scaphoid facet definition and possess radioulnarly narrow distal facets with no hamatolunate contact. Other groups lie intermediate between these two extremes (Figures 4 and S10). PC2 distinguishes (1) brachiators from all other locomotor behaviors (all *p* < 0.0001), (2) digitigrade taxa from of all other locomotor behaviors (all *p* < 0.0001), and (3) quadrumanous taxa from all other locomotor behaviors (all *p* < 0.0001). PC2 fails to distinguish among bipeds, knuckle walkers, and palmigrade taxa (all *p* = 1; Table S2).

#### 3.1.3 Triquetrum

The first 20 PCs explain 85.2% of variance in the triquetrum sample, PCs 1–4 explain 53.1%, and PCs 1 and 2 explain 39.8%.

PC1 (29.0% of variance) reflects (1) proximodistal and dorsopalmar dimensions and (2) overall ulnar side morphology, with palmigrade and digitigrade taxa at one extreme, possessing dorsopalmarly deep triquetra with ulnar articulation and large, proximally oriented pisiform facets and quadrumanous taxa at the other, possessing proximodistally long triquetra with small, palmarly oriented pisiform facets (Figures 5 and S11). PC1 distinguishes (1) brachiators from all other locomotor groups (all *p* < 0.0001) and (2) quadrumanous taxa from all other locomotor groups (all *p* < 0.0001). PC1 fails to distinguish between knuckle walkers and bipeds (*p* = 1) or between palmigrade and digitigrade taxa (*p* = 0.15; Table S3).

PC2 (10.8% of variance) reflects (1) the shape of the ulnodistal extent of the bone and (2) lunate facet curvature, with quadrumanous and some palmigrade and digitigrade specimens at one extreme, possessing a pointed, nonarticular ulnodistal extension and convex lunate facets and some knuckle walking and bipedal specimens at the other, lacking ulnodistal protuberances and possessing concave lunate facets (Figures 5 and S11). PC2 distinguishes (1) bipeds from all other locomotor behaviors (all *p* < 0.0001), (2) brachiators from all other locomotor behaviors (all *p* < 0.0001), (3) digitigrade taxa from all other locomotor behaviors (all *p* < 0.0001), and (4) knuckle walkers from all other locomotor behaviors (all *p* < 0.0001). PC2 fails to distinguish between quadrumanous and palmigrade taxa (*p* = 0.09; Table S3).

#### 3.1.4 Trapezium

The first 20 PCs explain 86.5% of variance in the trapezium sample, with PCs 1–3 explaining 51.1%, and PCs 1 and 2 explaining 43.1%.

PC1 (29.6% of variance) reflects (1) proximodistal and dorsopalmar dimensions and (2) tubercle size, with knuckle walkers and brachiators possessing proximodistally short, dorsopalmarly deep trapezia with large tubercles whereas the quadrumanous, palmigrade, and digitigrade groups possess proximodistally long, dorsopalmarly shallow trapezia with small tubercles (Figures 6 and S12). PC1 distinguishes (1) brachiators from all other locomotor behaviors (all *p* < 0.0001), (2) digitigrade taxa from all other locomotor behaviors (all *p* < 0.0001), (3) palmigrade taxa from all other locomotor behaviors (all *p* < 0.0001), and (4) quadrumanous taxa from all other locomotor behaviors (all *p* < 0.0001). PC1 fails to distinguish between bipeds and knuckle walkers (*p* = 1; Table S4).

PC2 (13.5% of variance) reflects first metacarpal facet curvature and palmar extent, with bipeds possessing large, palmarly expanded, saddle‐shaped first metacarpal facets whereas brachiators possess radially convex first metacarpal facets (Figures 6 and S12). PC2 distinguishes (1) bipeds from all other locomotor behaviors (all *p* < 0.0001), (2) brachiators from all other locomotor behaviors (all *p* < 0.0001), (3) and palmigrade taxa from all other locomotor behaviors (all *p* < 0.0001). PC2 fails to distinguish digitigrade taxa from knuckle walkers (*p* = 1) or quadrumanous (*p* = 0.89) taxa, although it distinguishes between knuckle walkers and quadrumanous taxa (*p* = 0.04; Table S4).

#### 3.1.5 Trapezoid

The first 20 PCs explain 88.1% of variance in the trapezoid sample, PCs 1–3 explain 52.0%, and PCs 1 and 2 explain 43.5%.

PC1 (27.9% of variance) reflects (1) dorsopalmar depth, (2) palmar beak shape, and (3) angle/orientation between second metacarpal facets with bipeds possessing dorsopalmarly deep trapezoids with more planar, distally oriented second metacarpal facets and radioulnarly broad palmar beaks at one extreme, and brachiators at the other, possessing dorsopalmarly shallow trapezoids with more mediolaterally oriented second metacarpal facets and extremely tapered palmar beaks resulting from acutely angled scaphoid and trapezium facets (Figures 7 and S13). PC1 distinguishes (1) bipeds from all other locomotor behaviors (all *p* < 0.0001) and (2) brachiators from all other locomotor behaviors (all *p* < 0.0001). PC1 fails to distinguish between knuckle walking and quadrumanous taxa (*p* = 1) or between palmigrade and digitigrade taxa (*p* = 1; Table S5).

PC2 (15.6% of variance) reflects scaphoid facet radioulnar extent, with significant overlap across many locomotor groups (Figures 7 and S13). PC2 distinguishes (1) brachiators from all other locomotor behaviors (all *p* < 0.0001) and (2) digitigrade taxa from all other locomotor behaviors (all *p* < 0.0001). PC2 fails to distinguish between knuckle walkers and bipeds (*p* = 1) and between bipedal and quadrumanous taxa (*p* = 0.17; Table S5).

#### 3.1.6 Capitate

The first 20 PCs explain 83.8% of the variance in the capitate sample, PCs 1–5 explain 55.6%, and PCs 1 and 2 explain 32.0%.

PC1 (17.8% of variance) reflects bipedal humans possessing radioulnarly wide capitate heads and necks, other extant non‐hominine taxa possessing radioulnarly narrow, “waisted” necks and heads, and knuckle‐walking African apes intermediate between these two extremes with increased head width (Figures 8 and S14). PC1 distinguishes (1) bipeds from all other locomotor groups (all *p* < 0.0001), (2) knuckle walkers from all other locomotor groups (all *p* < 0.0001), and (3) digitigrade taxa from all other locomotor behaviors (*p* < 0.0001 for all pairwise comparisons; Table S6).

PC2 (14.2% of variance) reflects hominoids possessing dorsopalmarly flat but topographically complex third metacarpal facets and palmarly restricted second metacarpal facets and non‐hominoids possessing dorsopalmarly “cupped” but simple third metacarpal facets and second metacarpal facets spanning the entire dorsopalmar length of the bone (Figures 8 and S14). PC2 distinguishes bipeds from all other locomotor behaviors (all *p* < 0.0001) but fails to distinguish between quadrumanous, knuckle walking, and brachiating taxa (all *p* = 1), and between digitigrade and palmigrade taxa (*p* = 1; Table S6).

#### 3.1.7 Hamate

The first 20 PCs explain 87.6% of the variance in the hamate sample, with PCs 1 and 2 accounting for 52.2% of the variance.

PC1 (31.3% of variance) reflects (1) proximodistal and radioulnar dimensions, (2) hamulus proximodistal orientation, and triquetral facet shape, with hylobatids possessing proximodistally long hamates with distally oriented hamuli and entirely convex triquetral facets whereas bipeds and some knuckle walkers (*Gorilla*) possess radioulnarly wide hamates with palmarly oriented hamuli and concavoconvex triquetral facets (Figures 9 and S15). PC1 distinguishes (1) bipeds from all other locomotor groups (all *p* < 0.0001) and (2) brachiators from all other locomotor groups (all *p* < 0.0001). PC1 fails to distinguish between digitigrade and knuckle‐walking taxa (*p* = 0.2) and between digitigrade, palmigrade, and quadrumanous taxa (all *p* = 1; Table S7).

PC2 (20.9% of variance) reflects (1) hamulus size and (2) dorsopalmar curvature of the metacarpal facets. At one extreme lies knuckle walkers and brachiators, with large hamuli and dorsopalmarly flat metacarpal facets. At the other extreme lies the quadrumanous, palmigrade, and digitigrade groups, with small hamuli and highly dorsopalmarly concave metacarpal facets (Figures 9 and S15). PC2 distinguishes (1) bipeds from all other locomotor behaviors (all *p* < 0.0001) and (2) quadrumanous specimens from those of all other locomotor behaviors (all *p* < 0.0001). PC2 fails to distinguish between knuckle walkers and brachiators (*p* = 1) and between digitigrade and palmigrade taxa (*p* = 0.11; Table S7).

### 3.2 Euclidean distances

#### 3.2.1 Scaphoid

**FIGURE 10:**
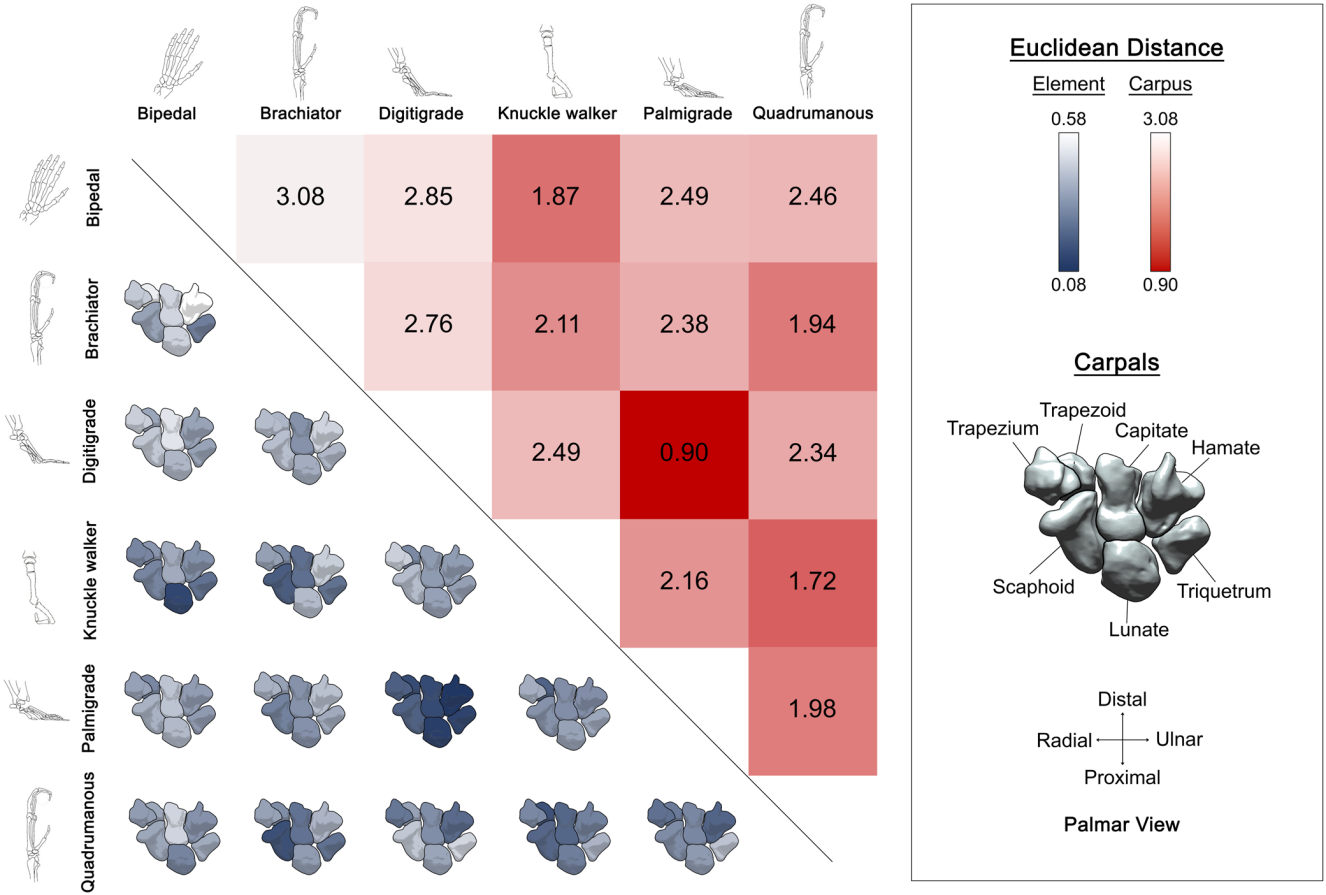
Pairwise comparisons between all locomotor behaviors, calculating mean Euclidean distances for each individual carpal element (blue) and for the entire carpus as a unit (red). Darker colors indicate greater similarity. Articulated carpus in palmar view is a *Gorilla gorilla* (specimen KUPRI 23), with carpals labeled on the right.

The Euclidean distances between brachiator, knuckle walking, and quadrumanous groups are small, suggesting morphological similarity when the scaphoid and centrale are digitally fused in quadrumanous and brachiator taxa (Figure 10; Table S8). Likewise, there is low Euclidean distance between palmigrade and digitigrade taxa, although the scaphoid shows the greatest Euclidean distances among the carpals between these two groups (Figure 10; Table S8).

#### 3.2.2 Lunate

The bipedal and brachiator groups possess the greatest Euclidean distances in lunate morphology and the palmigrade and digitigrade groups the smallest (Figure 10; Table S8). There are no locomotor groups with consistently higher Euclidean distances.

#### 3.2.3 Triquetrum

The Euclidean distances in triquetrum morphology between locomotor groups reflect the results of PC1, showing smaller distances between palmigrade and digitigrade taxa and between bipedal/brachiator/knuckle‐walking taxa (Figure 10; Table S8). The greatest Euclidean distance is between digitigrade and quadrumanous taxa and the lowest between palmigrade and digitigrade taxa (Figure 10; Table S8).

#### 3.2.4 Trapezium

The digitigrade and knuckle walking groups have the greatest Euclidean distances in trapezium morphology (Figure 10; Table S8). The Euclidean distance between palmigrade and digitigrade taxa is the lowest between all groups but high relative to other carpals' Euclidean distances between these two groups, second only to the scaphoid. Notably, the mean distance between all locomotor behaviors is markedly high but the variance in Euclidean distances across groups is low, suggesting that this element is particularly distinct across locomotor behaviors (Figure S17).

#### 3.2.5 Trapezoid

The Euclidean distances in trapezoid morphology between locomotor groups are generally low (Figure 10; Table S8), as reflected in this element possessing the lowest mean Euclidean distance across all locomotor groups pooled (Figure S17). The lowest Euclidean distance is between the palmigrade and digitigrade groups, and the highest is between brachiator and bipedal groups (Figure 10; Table S8).

#### 3.2.6 Capitate

**FIGURE 11:**
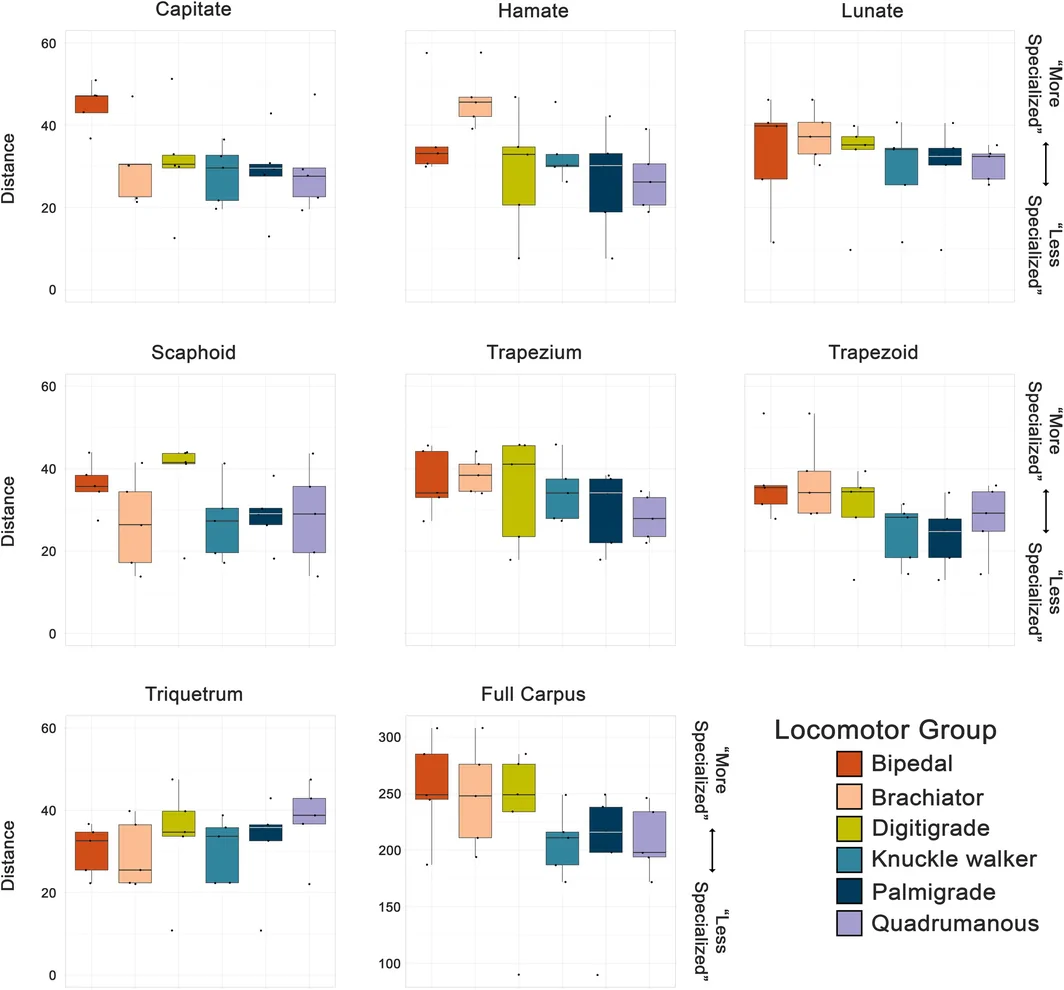
Box‐and‐whisker plots visualizing how distant each locomotor group is from all other locomotor groups, with each point representing the distance between two locomotor groups (e.g., each point in the left‐most box of the top left panel represents the morphological distance between the bipedal capitate and the capitate morphology of another locomotor group). Groups with higher distance values (e.g., the bipedal capitate) are more morphologically distinct (specialization Use Type 4; Table 1). Boxes represent first and third quartiles, whiskers extend 1.5× the interquartile range, horizontal lines represent the median of the sample distribution, and symbols represent individual data points.

The highest Euclidean distance in capitate morphology is between bipedal and digitigrade taxa, and the lowest was between palmigrade and digitigrade taxa (Figure 10; Table S8). Bipeds consistently have higher Euclidean distances in capitate morphology from all other locomotor groups than any other group has from each other (Figure 11).

#### 3.2.7 Hamate

Of all carpals investigated in the current study, the hamate possesses the most variance in Euclidean distances (Figure S16), with bipeds and brachiators showing the highest Euclidean distances and palmigrade and digitigrade taxa showing the lowest (Figure 11; Table S8). Like the capitate of bipeds, the hamate of brachiators consistently has higher Euclidean distances from all other locomotor groups than any other group has from each other (Figure 11). Palmigrade taxa display the least morphologically distinct hamate; although it is extremely close to that of digitigrade taxa, its Euclidean distances from other locomotor groups were consistently lower than those of the digitigrade hamate (Figure 11; Table S8).

#### 3.2.8 Full carpus

Euclidean distances in carpal morphology between each pair of locomotor groups reveal that palmigrade and digitigrade taxa by far have the most similar carpus (and individual carpal elements) to one another than any other pair (Figure 10; Table S8). Between these two groups, there is a trend in carpal similarity such that more radially located carpals (trapezium, scaphoid) are more dissimilar than more ulnarly located carpals (triquetrum, hamate). Bipeds tend to have the most distinct carpus relative to other locomotor groups, differing most from brachiators but most closely resembling that of knuckle walkers, a trend chiefly driven by moderate morphological similarity across all carpals and high lunate morphological similarity (Figure 10; Table S8). However, the carpus of knuckle walkers more closely resembles that of quadrumanous taxa rather than bipeds, driven by greater similarity in scaphoid, capitate, and trapezoid morphology (Figure 10; Table S8). Although brachiating and quadrumanous taxa share a tensile and vertical wrist posture, the quadrumanous carpus is more like that of knuckle walkers than brachiators but resembles that of brachiators approximately as much as it resembles that of palmigrade taxa. The carpus of palmigrade taxa is generally intermediate in morphology between the digitigrade carpus and those of all other locomotor groups. Therefore, notably, despite the high similarity between the digitigrade and palmigrade groups and despite the shared compressive vertical hand posture of digitigrady and knuckle walking, the digitigrade group emerges as one with relatively overall high morphological distinctiveness or distance from other groups (Figure 11). Among hominoids, the carpus of quadrumanous taxa showed the most similarities to that of palmigrade and digitigrade taxa, owing mostly to trapezium and hamate morphology and resulting in the lowest full carpus overall morphological distinctiveness (Figures 10 and 11; Table S8).

## 4 DISCUSSION

### 4.1 Many purported knuckle‐walking traits are found in nonhuman anthropoids or hominoids, suggesting they are primitive in African apes

By investigating a broader anthropoid sample that includes non‐hominid taxa, the current study tested whether purported knuckle‐walking traits are shared with other primate taxa, indicating that they are not shared, derived characteristics in response to the biomechanical demands of knuckle walking. Ultimately, our analyses of carpal morphology were unable to detect any morphological feature that is found in all knuckle‐walking taxa to the exclusion of all other locomotor groups. Instead, several purported knuckle‐walking carpal features clearly represent morphology shared among either anthropoids broadly, hominoids broadly, or the African ape‐human clade specifically. For example, there is a general pattern of proximodistally short carpals (Table 3, Trait 12) in knuckle walkers relative to those of brachiators and quadrumanous taxa, but the same pattern is broadly observed in digitigrade relative to palmigrade taxa, suggesting that this is common to primates that compressively load a vertical manus.

**TABLE 3:** Results of current study's investigation of distribution of purported knuckle‐walking traits (visualized in Figure 1), listing which extant taxa possess each trait, the proposed character polarity, and proposed alternative function of these traits based on the results of this study.

	Trait	Taxa with trait	Proposed polarity	Proposed alternative function
1	Complex second metacarpal articular facet on trapezoid and capitate^1^	*Pongo*, hylobatids, *Pan, Gorilla*	Derived complex facet in hominoids, secondarily derived planar facet in Homo OR derived complex facet only in hylobatids, *Pongo, Pan*, *Gorilla*	Resists shear stresses in pronation and supination of the metacarpals during below‐branch suspension^2^
2	Radioulnarly narrow capitate neck^3^	*Sapajus*, cercopithecoids, hylobatids, *Pongo, Pan, Gorilla*	Derived wide capitate neck in Homo	Wide capitate neck in Homo dissipates palmodorsal loads during tool use,^4^ accommodates larger, more palmarly placed trapezoid facet
3	Radially expanded capitate head^5^	*Pan, Gorilla, Homo*	Derived radially expanded capitate head in hominines	Limits extension at midcarpal joint in screw‐clamp mechanism^6^ persisted in Homo OR evolved in response to scaphoid–centrale fusion
4	Proximally placed ridge on distal extent of lunate facet on capitate^1^	*Sapajus*, cercopithecoids, hylobatids, *Pan, Gorilla, Homo*	Derived distal placement in Pongo	Distally placed distal extent of lunate facet on Pongo capitate emerges from large Pongo lunate
5	Scaphoid–centrale fusion^7^	*Pan, Gorilla, Homo*	Derived in homininines	Limits extension at midcarpal joint in screw‐clamp mechanism^6^ OR developed in hominines stochastically
6	Ridge at boundary between radius and trapeziotrapezoid facets on scaphoid^8^	*Pan, Gorilla*. When scaphoid and centrale artificially fused: *Sapajus*, cercopithecoids, hylobatids, *Pongo*	Derived scaphoid–centrale fusion in hominines, derived absence of ridge in Homo	Absence of ridge at boundary between radius and trapeziotrapezoid facets on Homo scaphoid emerges to accommodate wide capitate neck and/or in response to opening of capitate facet
7	Presence of large articular facet for hamate on lunate^9^	Hylobatids, *Pongo, Pan, Gorilla, Homo*	Derived in hominoids	Facilitates pronation and supination at midcarpal joint during below‐branch suspension^10^
8	Concavoconvex, “spiral” triquetral facet on hamate^11^	*Sapajus*, cercopithecoids, *Pongo, Pan, Gorilla, Homo*	Derived reduction/absence of concavity at distal extent in hylobatids	Absence of concavity at distal extent of hylobatid triquetral facet on hamate facilitates pronation and supination at midcarpal joint during brachiation^10^
9	Proximally placed ridge on distal extent of the hamate's articular facets with the proximal carpal row^1^	Cercopithecoids (variable), *Pan, Gorilla*	Unclear	No proposed alternative function
10	Keel between fourth and fifth metacarpal facets on hamate^12^	Not recovered	No proposed polarity	No proposed alternative function
11	Larger fourth metacarpal facet than fifth metacarpal facet on hamate^13^	Not recovered	No proposed polarity	No proposed alternative function
12	Proximodistally short carpals^14^	Continuous. In order from longest to shortest: hylobatids, *Pongo*, most cercopithecoids and Sapajus, *Papio* and *Erythrocebus, Pan, Gorilla, Homo*	Derived in digitigrade taxa, derived in hominines convergently OR derived proximodistal lengthening in hylobatids and Pongo	Resists bending forces during proximodistally oriented compressive loading^15^ AND/OR proximodistally long carpals in Pongo and hylobatids increase below‐branch suspensory stride length^16^

^1^ Orr (2005); Richmond (2006); Richmond et al. (2001); Tuttle (1967); Jenkins and Fleagle (1975).

^2^ Selby et al. (2016); Lovejoy, Simpson, et al. (2009).

^3^ Clark (1947); Lewis (1972, 1973); Tuttle (1967).

^4^ Marzke (1983).

^5^ Lewis (1985).

^6^ Conroy and Fleagle (1972); Corruccini (1978); Dainton and Macho (1999); MacConaill (1941); Orr (2018).

^7^ Conroy and Fleagle (1972); Corruccini (1978); Dainton and Macho (1999); MacConaill (1941); Marzke (1971); Orr (2018); Püschel et al. (2020); Richmond et al. (2001); Tuttle (1967).

^8^ Richmond et al. (2001); Richmond and Strait (2000); Tuttle (1967).

^9^ Bain et al. (2015); Marzke et al. (1994); Sarmiento (1988).

^10^ Jenkins and Fleagle (1975).

^11^ Lewis (1972); Lewis (1973); Richmond et al. (2001).

^12^ Richmond et al. (2001).

^13^ Marzke et al. (1994); Tuttle (1967).

^14^ Dainton and Macho (1999); Inouye (1994).

^15^ Goldstein and Sylvester (2023).

^16^ Drapeau and Ward (2006).

**FIGURE 12:**
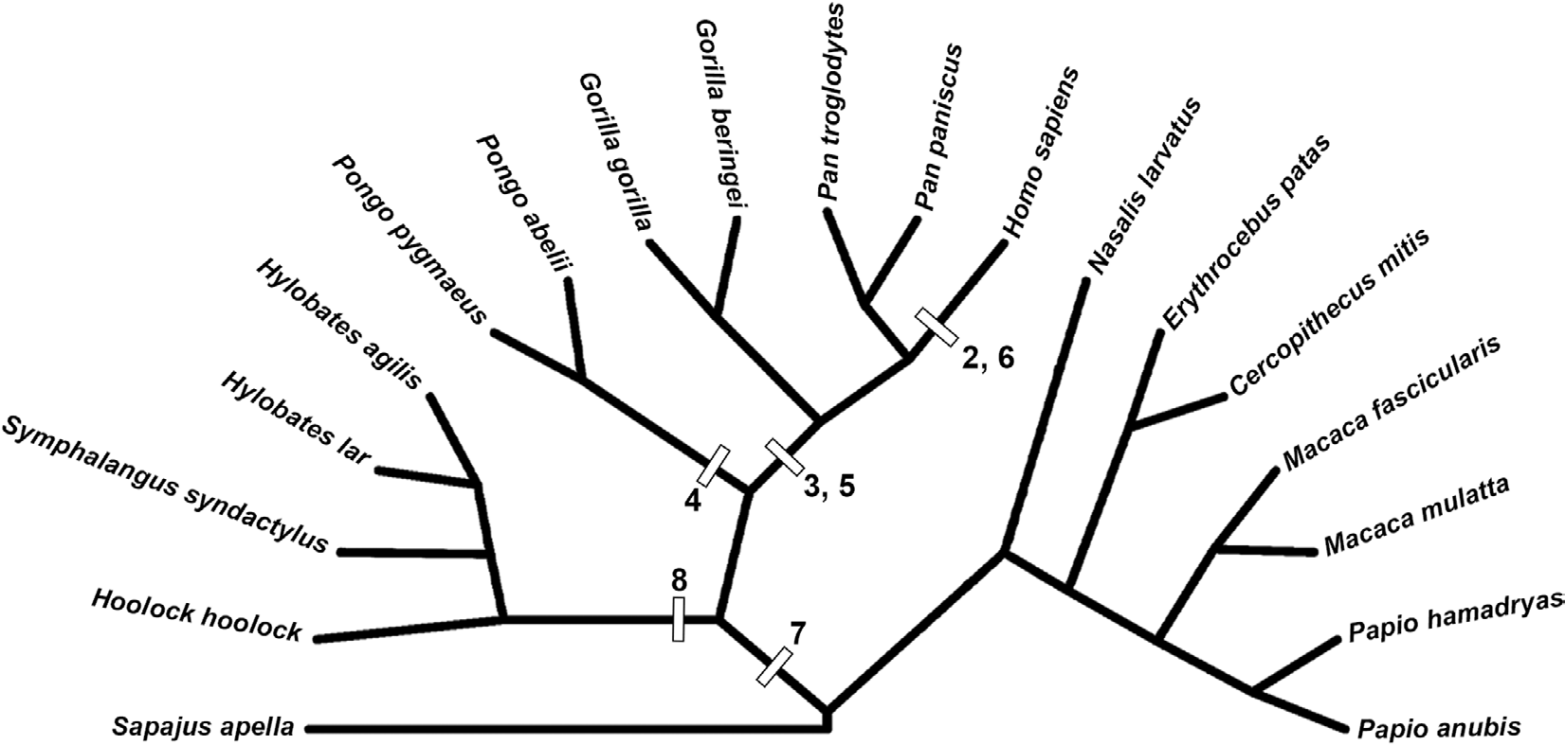
Phylogenetic tree with the locations of proposed trait **changes** mapped. Traits with uncertain polarities (traits 1 and 10–12) are omitted. See Table 3 for further description.

Other features previously associated with proximodistal compressive loading in knuckle‐walking taxa specifically were not recovered by SPHARM analysis at all. For instance, no signal associated with differences in the relative sizes of the fourth and fifth metacarpal facets on the hamate was recovered (Table 3, Trait 11), nor any signal related to the size or presence of a keel between these two facets (Table 2, Trait 10). It is possible that these features were too subtle for SPHARM to detect on the hamate, which shows substantial overall morphological variation (Figures 9 and S17). Differences in relative fourth and fifth metacarpal facet sizes have been quantified in analyses that focus landmarks on this particular region of the hamate (Almécija et al., 2015; Daver et al., 2014; Vanhoof et al., 2021), suggesting this trait may, in fact, be too subtle for SPHARM to detect. Close visual anatomical study of the sample shows substantial intraspecific variation in the presence of a keel between the fourth and fifth metacarpal facets, with a frequent absence in knuckle‐walking taxa and presence in non‐knuckle‐walking taxa including hylobatids and macaques. This variation may explain why SPHARM analysis failed to identify a clear signal for this trait. Our analysis also found no evidence of a more proximally placed, raised distal margin of the lunate facet on the dorsal hamate in knuckle‐walking relative to other taxa (Table 3, Trait 9). Studies proposing this as a knuckle‐walking trait likewise recovered substantial variation across cercopithecoid taxa and suggested that this trait may be more predictive in the capitate than it is in the hamate (Richmond et al., 2001). As such, variation may also play a role in the absence of a clear signal associated with this trait in the current study. Our analysis supports the claim made previously (Kivell & Schmitt, 2009) that the location and morphology of the dorsodistal margin of the hamate's articular facets for the proximal carpal row are not diagnostic. Similarly, our analysis found that the distal margin of the lunate facet on the capitate (Table 3 and Figure 12, Trait 4) does not appear to be uniquely proximal in knuckle walkers. Instead, it appears variable but common among anthropoids for the lunate facet to terminate at the approximate center of the capitate's proximodistal length (but see Orr, 2017 for an alternative method of measurement). *Pongo* taxa appear to be the exception to the general anthropoid condition, separating from all other taxa on PC4, which is associated with a more distal termination of this facet (Figure S16).

Other knuckle‐walking traits that appear to represent the common anthropoid condition include (1) the presence of a ridge between the trapeziotrapezoid and radius facets of the scaphoid (Table 3 and Figure 12, Trait 6) and (2) a concavoconvex triquetrum facet on the hamate (Table 3 and Figure 12, Trait 8). Identification of this ridge on the scaphoid has been complicated by the absence of scaphoid–centrale fusion in non‐hominine taxa. This ridge is located where the scaphoid and centrale parts of the fused hominine scaphoid meet dorsodistally and so absence of fusion between these two elements makes it difficult to discern whether a ridge “should” be present at the junction between these two bones. Because the current study digitally fused the scaphoid and centrale together, it was possible to investigate this feature in non‐hominine taxa and determine whether the presence of this ridge occurs only in knuckle‐walking taxa or its absence only occurs in *H. sapiens*. The PCA of scaphoid–centrale morphology shows that the latter is correct (Figure 3). The emergence of this feature in nonhominines with digitally fused scaphoid–centrales suggests that this ridge simply emerges as a by‐product of scaphoid–centrale fusion. This does not preclude the biomechanical significance of this ridge for limiting midcarpal extension during knuckle walking, but it is a consequence of the scaphoid–centrale fusing and thus not an independent trait. The absence of this ridge in *H. sapiens* may have evolved to accommodate a larger trapezium facet on the scaphoid as the radial wrist underwent selection for a larger trapezium. Alternatively, the ridge may have been obliterated as hominins evolved an open capitate facet of the scaphoid to accommodate a wider capitate neck.

The concavoconvex facet for the triquetrum on the hamate (Table 3 and Figure 12, Trait 8) also appears to be common to all anthropoids except brachiators. One of many features that separates brachiators from all other taxa along PC1 for the hamate is the large, convex triquetral facet of brachiators in contrast to the concavoconvex facet present in all nonbrachiating taxa (Figures 9 and S15). The presence of this facet morphology in most anthropoid taxa suggests that the general concavoconvex form is ancestral and that brachiators underwent selection for an extreme, “ball‐like” triquetral facet morphology, likely related to increased wrist pronation–supination during brachiation (Jenkins, 1981). As is the case with the ridge separating the trapeziotrapezoid and radius facets on the scaphoid, it is possible that this concavoconvex facet morphology is biomechanically advantageous for knuckle walking, but the difference between knuckle‐walking taxa and others appears to be one of degree but not kind.

Some knuckle‐walking traits are present in most or all hominoid taxa. The complex second metacarpal articular facets on the trapezoid (Table 3, Trait 1) unites all hominoids along PC2 (Figure 7). Likewise, the complex third metacarpal articular facets on the capitate (Table 3, Trait 1) unite all non‐*Homo* hominoids along PC2 (Figure 8), with more suspensory taxa (brachiator, quadrumanous) possessing more positive values, associated with greater third metacarpal facet complexity (Figures 8 and S14). This result is consistent with claims that this feature and corresponding complexity on the third metacarpal's capitate facet is associated with resisting torsion and shear stresses imposed on the forelimb during below‐branch suspension (Lovejoy, Simpson, et al., 2009; Rein, 2019; Selby et al., 2016) and refutes suggestions that third metacarpal facet complexity evolved in hominines to stabilize the metacarpal against sliding on the capitate during weight‐bearing (Marzke & Marzke, 2000). The fact that *H. sapiens*—the only hominoid that does not typically include below‐branch suspension in its locomotor repertoire—lacks third metacarpal facet complexity lends further support to this interpretation.

In fact, *H. sapiens* have the most distinct capitate morphology of all anthropoid taxa sampled, primarily driven by the radioulnarly wide capitate head and neck, which separates them from all other taxa along PC1 (Figure 8). This finding has implications for how one interprets the capitate waisting character complex because it demonstrates that all other taxa possess at least one of the capitate waisting features: a radioulnarly narrow capitate neck (Table 3 and Figure 12, Trait 2; O'Connor, 1975; Corruccini, 1978). However, our results also confirm quantitatively the assertion of Lewis (1985) that cercopithecoids lack radial expansion of the capitate head (Table 3 and Figure 12, Trait 3) and instead achieve this “waisted” appearance via radioulnar expansion of the distal end and an ulnarly directed tilt of the capitate head relative to the rest of the bone. This can be seen along PC2 of the capitate, in which negative values unite palmigrade and digitigrade cercopithecoids and *Sapajus* based on these two features (Figure 8). This analysis thus reveals that one feature of capitate waisting (a radioulnarly narrow capitate neck; Table 3 and Figure 12, Trait 2) is present in most anthropoids. The other (a radially expanded capitate head; Table 3 and Figure 12, Trait 3) can be interpreted as present in both bipeds and knuckle walkers; *H. sapiens* separate from other taxa along PC1 because both the capitate head and neck are radioulnarly as wide as the distal extent of the capitate, whereas knuckle‐walking taxa lie intermediate along this axis (Figure 8), reflecting a distinct combination of these two traits: a radially expanded capitate head with a radioulnarly narrow capitate neck. Separating this composite feature into these two traits reveals that, although knuckle‐walking taxa possess a distinct combination of these traits, neither trait alone is distinct. This finding is consistent with previous descriptions of hominine capitate morphology (Kivell & Schmitt, 2009; Lewis, 1972, 1973) and indicates that there is no single feature of capitate waisting that is shared by all knuckle‐walking taxa and is absent in all nonknuckle‐walking taxa.

Morphological similarities among cercopithecoids, *Pan*, and *Gorilla* absent in other hominoids could be interpreted as convergences because of the biomechanical similarities of digitigrady and knuckle walking, chiefly the limited extension seen during these hand postures and the proximodistally oriented compressive mechanical loads incurred on the wrist. However, forelimb kinematic studies on digitigrade monkeys demonstrate that these taxa are capable of wrist extension comparable to that of palmigrade taxa (Patel, 2009, 2010b; Patel & Wunderlich, 2010; but see Tuttle, 1969; Orr, 2017), and so any carpal morphological similarities between knuckle walking and digitigrade taxa appear unrelated to limiting wrist extension. These findings lend support to the hypothesis that the limited wrist extension observed in knuckle‐walking taxa arises from ligamentous and muscular—as opposed to osteological—features of the forelimb.

The remaining biomechanical similarity between digitigrady and knuckle walking —a proximodistally loaded vertical manus—is not present in palmigrady, but the current study finds that all purported knuckle‐walking traits shared between digitigrade knuckle‐walking taxa (except for reduced proximodistal length of carpals) are also present in palmigrade anthropoids, corroborating the findings of Kivell and Schmitt (2009). As such, these shared features must not reflect convergence associated with either of the chief biomechanical similarities between digitigrade and knuckle‐walking taxa. An alternative biomechanical interpretation of the features shared across knuckle walking, palmigrade, and digitigrade taxa is that they are related to compressively loading the forelimb regardless of wrist/hand posture or—as argued by Kivell and Schmitt (2009) – when the wrist is even slightly extended. This is incongruent with the initial functional interpretations of these features as related to limiting wrist extension, and the extended versus columnar hand posture distinction is predicated on the hypothesis that the *Gorilla* knuckle‐walking posture is more columnar than that of *Pan*, which does not appear supported by biomechanical studies (Finestone et al., 2018; Thompson, 2020).

The numerous features previously associated with knuckle walking that are found in all hominoids may more likely be associated with the evolution of vertical climbing or below‐branch suspension. Features found in all hominoids except for *Homo* can be interpreted similarly, with *Homo* having lost these traits as the hominin lineage became increasingly bipedal and thus relied less on these arboreal behaviors. Put simply, our results suggest that the character polarity of several putative knuckle‐walking features must be called into question and that the knuckle‐walking carpus may, in fact, represent a more generalist hominoid wrist in contrast to the specialized wrists—or individual carpals—of other apes. Furthermore, this apparent dearth of clear knuckle‐walking anatomical specializations (**Use Type 2**) unique to *Pan* and *Gorilla* may provide an explanation for the lack of clear knuckle‐walking specializations reported in *Ardipithecus ramidus* (Lovejoy, Simpson, et al., 2009). In fact, the current study finds that many of the purported knuckle‐walking specializations reported absent in *Ardipithecus ramidus* may more accurately be characterized as suspensory specializations given their presence in all suspensory taxa included in the current study's sample. Evaluating the evolution of suspensory behavior in hominoids, however, is outside of the scope of this study.

It should be noted that the interpretation of putative knuckle‐walking traits as ancestral anthropoid traits or hominoid synapomorphies does not preclude the functional relevance of these features to knuckle walking but rather calls into question whether these features are locomotor adaptations selected for knuckle walking or rather ancestral morphologies exapted for knuckle walking. That is to say, all of the purported knuckle‐walking features investigated in the current study may be *functionally* important for knuckle walking even though they may not have *evolved* as adaptations for knuckle walking. This functional evolutionary distinction of how one defines a “knuckle‐walking feature” is an important one, as debate and/or confusion surrounding the evolution of knuckle walking may arise due to the assumption that a functional feature must necessarily be an evolutionary one, which arose via selection in response to functional demands rather than repurposed. This is particularly relevant to joint functional complexes such as the carpus, in which multiple elements are in direct physical contact with one another and likely share several developmental pathways (Anaya & Boyer, 2026; Okamoto et al., 2026) and thus each element's morphology is influenced by the morphology of the others. This may impose constraints on each element's evolution and/or increase the frequency of exaptations or parallel evolution. Future research on correlated evolution and development of these elements to shed further light on this possible phenomenon is warranted, especially in light of metric analyses of the capitate and third ray that did not recover any meaningful pattern of integration (Williams, 2010).

### 4.2 Possible support for a broadly African ape‐like carpus in the panin–hominin last common ancestor?

To confidently assert that *Pan* and *Gorilla* (and thus *Homo*) share a single knuckle‐walking origin, two criteria must be met: (1) either *Pan* and *Gorilla* or all three extant hominine genera share some derived traits of carpal morphology and (2) these synapomorphies arose as adaptations for knuckle walking. Here, we discuss whether or not the results in the present study meet these two criteria. An interpretive challenge with the first of these criteria is that the presence of shared, derived traits in *Pan* and *Gorilla* but not *Homo* may indicate convergent origins but also could have arisen from a single knuckle‐walking origin with secondary loss of these traits in hominins. Furthermore, the presence of shared, derived hominine traits in *H. sapiens*—which does not knuckle walk—could suggest that these traits are unrelated to knuckle walking or may have persisted in the hominin lineage due to phylogenetic lag or because they were co‐opted for other activities, such as tool use. The current study did not observe any shared, derived traits present in only *Pan* and *Gorilla*, but there appear to be some traits evaluated here which are shared between *Pan, Gorilla*, and *Homo*. If one could confidently ascertain that these traits arose as adaptations to knuckle walking, this would present possible evidence that knuckle walking evolved once in the common ancestor of hominines.

These shared traits include (1) radial expansion of the capitate head (Richmond, 2006; Table 3 & Figure 12, Trait 3), (2) fusion of the scaphoid–centrale (Kivell & Begun, 2007); Table 3 and Figure 12, Trait 5), and (3) proximodistally short carpals relative to those of particularly brachiating and quadrumanous taxa (Table 3, Trait 12). Previous work investigating the development of scaphoid–centrale fusion (Kivell & Begun, 2007) supports this finding and suggests that scaphoid–centrale fusion is derived in hominines. In addition to these shared traits, the PCA and Euclidean distance results show that lunate morphology in particular is strikingly similar across all hominines (Figures 4 and 11), a result that may explain why hominoid lunate morphology remains relatively understudied compared with other bones of the wrist (Corruccini, 1978; Schwartz & Yamada, 1998; Dainton & Macho, 1999; Ward et al., 1999; Jenkins & Fleagle, 1975; Kivell et al., 2013). One should note that these results are in contrast with research investigating the evolution of lunate proportions that found that *Pan* and *Pongo* are more similar (Kivell et al., 2013). However, the current study evaluates aspects of morphology beyond proportion and thus appears to capture additional subtleties in lunate morphology that have previously only been described qualitatively (e.g., differences in scaphoid facet placement between hominines and other taxa (Begun, 1994) or in triquetrum facet curvature and orientation (Orr et al., 2023; Sarmiento, 1988).

It is notable that the current study recovered traits of the capitate, scaphoid, and lunate which could be hominine synapomorphies, as these three carpals are involved in the screw‐clamp mechanism, a proposed knuckle‐walking adaptation in which the proximal and distal elements of the radial‐side wrist “lock” into place during extension, thus limiting wrist range of motion and preventing it from collapsing during knuckle walking (MacConaill, 1941; Lewis, 1973; Jenkins & Fleagle, 1975; Orr, 2018). This occurs through supination of the scaphoid during extension, causing the distal edge of its capitate facet to rapidly engage with the capitate's neck. This rotation simultaneously brings the scaphoid closer to the lunate while the triquetrum supinates on the hamate, effectively pinning the lunate in place. More research testing the functional significance of the screw‐clamp mechanism in knuckle walking is warranted. However, detailed cineradiographic studies of wrist extension in *Pan*, *Pongo*, and select cercopithecoids observe the screw‐clamp mechanism in *Pan* and demonstrate that the *Pan* carpus reaches a “close‐packed” relationship between the scaphoid, capitate, and lunate at a lower degree of extension than other taxa caused by the screw‐clamp mechanism described above (Orr, 2017, 2018; Orr et al., 2010).

The chief morphological features implicated in the screw‐clamp mechanism are scaphoid–centrale fusion and capitate “waisting,” which includes a narrow capitate neck and radial expansion of the capitate head. *H. sapiens* possess a derived wide capitate neck that is hypothesized to allow for greater midcarpal extension than in African apes (Lewis, 1985), but the current study finds that the other two chief features of the screw‐clamp mechanism (scaphoid–centrale fusion and radial expansion of the capitate head) are shared, derived traits present in *Pan, Gorilla*, and *Homo*. Furthermore, their presence in early hominins supports the hypothesis that these traits did not independently emerge in these three taxa (Bush et al., 1982; Clark, 1947; Kivell, Churchill, et al., 2018; Lovejoy, Simpson, et al., 2009; Simpson et al., 2019; Ward et al., 1999) and that early hominin wrist kinematics included the screw‐clamp mechanism. However, although existing kinematic studies suggest that the screw‐clamp mechanism limits wrist extension in taxa with scaphoid–centrale fusion and capitate waisting (Orr, 2017, 2018; Orr et al., 2010), more research is necessary to ascertain the magnitude of its effect. That is to say, the presence of these putative screw‐clamp mechanism traits in hominins suggests a relative reduction in midcarpal extension, but it is unclear how much impact this would have had on overall wrist extension and whether other hard or soft tissue traits such as those suggested to increase midcarpal extension in *Ardipithecus* ramidus (Lovejoy, Simpson, et al., 2009) may have compensated for the limiting role of the screw‐clamp mechanism.

Although largely correlative, the involvement of the lunate in the screw‐clamp mechanism as a structure pinned between surrounding elements of the proximal carpal row and a striking similarity in lunate morphology among hominines may suggest that the morphology of the lunate could be important in the screw‐clamp mechanism. Further research investigating other possible explanations is warranted, but knuckle walking remains the most well‐supported hypothesis for the biomechanical significance of these features in concert with each other (Kivell & Begun, 2007; Orr, 2018; Püschel et al., 2020), thus suggesting a shared knuckle‐walking origin.

That said, although knuckle walking is the most well‐supported biomechanical hypothesis to explain these features, the supporting evidence is not strong, and an exploration of alternative hypotheses is warranted. Because it is a composite trait, capitate waisting has been defined and/or quantified in several ways (Corruccini et al., 1975; Lewis, 1985; Richmond, 2006; Kivell & Schmitt, 2009), with head radial expansion absent from some definitions (Corruccini et al., 1975; Kivell & Schmitt, 2009). This has led some studies to conclude that the capitates of cercopithecoids are waisted (Corruccini et al., 1975) and/or that it is more consistently present and visually prominent in *Pan* than in *Gorilla* (Kivell & Schmitt, 2009). Our results are consistent with the second of these conclusions; in this sample, capitate head radial expansion is rarely present in other taxa (e.g., *Pongo*) and often present in *Gorilla* but is only consistent—particularly in the anterior portion—in *Pan* and *Homo*. This calls into question the biomechanical necessity of capitate head radial expansion in knuckle walking.

Similarly, although scaphoid–centrale fusion appears to have emerged at the base of the hominine clade and is *functionally* involved in the screw‐clamp mechanism, it is impossible to determine whether this developmental difference *evolved* in response to selective pressures to diffuse forces and limit extension at the midcarpal joint during knuckle walking (Orr, 2018; Püschel et al., 2020) or simply arose stochastically and was subsequently exapted for knuckle walking. Because scaphoid–centrale fusion has emerged in numerous mammals that do not knuckle walk, including lemurs such as *Avahi*, *Indri*, and *Propithecus* (Jouffroy, 1975), it is possible that its emergence in hominines has little to do with knuckle walking. Likewise, the biomechanical role of lunate morphology in hominines remains underexplored, and it remains unclear whether its morphological similarities across hominines are due to their shared evolutionary history, stochastic processes, or a consequence of scaphoid–centrale fusion, which appears to have some bearing on the morphology of the mating articular facets of the scaphoid and lunate (Begun, 1994).

Regardless of whether they emerged as adaptations for knuckle walking, the presence of these shared, derived traits in *Pan, Gorilla*, and *Homo* suggests that the panin–hominin LCA possessed an African ape‐like carpus. This is further supported by the results of the Euclidean distances analyses conducted in the current study, which found that the wrist of *H. sapiens* is morphologically closer to those of knuckle‐walking African apes than any other group, both for all individual carpals and for the carpus as a whole. These results do not support the hypothesis that the panin–hominin LCA possessed a more primitive, Miocene ape‐like carpal morphology or that *Pan* and *Gorilla* carpal morphology converged.

### 4.3 The carpals of knuckle walkers are not too specialized to represent the ancestral hominin carpus

The Euclidean distances between the carpals of different locomotor groups quantitatively identified several morphologically distinct (**Use Type 4** or relationally specialized) elements, including the capitate of bipeds, the hamate of brachiators, and the scaphoid of digitigrade taxa (Figure 11). Furthermore, there is evidence that these elements have morphological traits that are reasonably hypothesized as “anatomical specializations” (**Use Type 2**) that evolved in response to increased or relaxed selective pressures to effectively perform certain tasks. One example of this is in the *H. sapiens* capitate, whose wide neck is hypothesized to help resist axial loads during percussive stone tool production (Marzke, 1983) and appears associated with expansion and migration of the capitate's trapezoid facet in response to other morphological changes to the radial wrist hypothesized to reduce tissue damage during percussive stone tool production (Tocheri, 2007; Tocheri et al., 2005). Another example is the morphologically distinct scaphoid of digitigrade taxa; the remarkably small tubercle and reduced dorsopalmar breadth of the digitigrade scaphoid may reflect relaxed selection on carpal tunnel breadth, as the extrinsic flexor muscles remain passive during digitigrady (Patel et al., 2012), unlike their active recruitment in palmigrady (Patel et al., 2012) or suspension (Susman & Stern, 1979).

In addition to results identifying individual carpal bones which appear relationally specialized, Euclidean distance analysis of the full carpus found that some locomotor or hand posture groups appear to possess a more morphologically distinct (i.e., relationally specialized per **Use Type 4**) carpus than others. Specifically, bipeds, brachiators, and digitigrade taxa each possess a carpus which is on average more distant in morphospace from those of other groups (Figure 11). This result indicates that these groups are particularly specialized (**Use Type 4**) among anthropoid primates and thus serve as particularly poor models for an ancestral condition such as the ancestral morphology of the hominin wrist. By contrast, palmigrade, knuckle walking, and quadrumanous taxa each possess a carpus which is on average less distant in morphospace from those of other groups (Figure 11). It is tempting to interpret this as evidence that these three groups thus equally serve as good models for an ancestral condition, but it should be noted that a limitation of this study is that it does not account for the nonindependence of phylogeny—and four of the six groups analyzed consist entirely of hominoids. Because closely related taxa tend to resemble each other, this likely had the effect of inflating the morphological distance values of two groups (digitigrade and palmigrade) and deflating the morphological distance values of the four hominoid groups (brachiator, quadrumanous, biped, knuckle walking). This suggests that the “specialization” signal of bipeds and brachiators is quite strong but also means that interpreting how well each of the less specialized groups (palmigrade, knuckle walking, quadrumanous) serve as models for an ancestral condition relative to each other should be approached with caution.

The finding that the carpus of bipeds (i.e., modern humans) is likely a poor representation of an ancestral carpus condition stands in contrast to the claim that the forelimbs of modern humans are primitive (Lovejoy, Simpson, et al., 2009). However, fossil evidence from several hominin species including *Australopithecus afarensis* (Bush et al., 1982; Johanson, Lovejoy, et al., 1982; Marzke, 1983; McHenry, 1983), *Au. africanus* (Broom & Schepers, 1946; Clark, 1947; McHenry, 1983), *Au. sediba* (Kivell, Churchill, et al., 2018), *Paranthropus boisei* (Mongle et al., 2025), and *H. floresiensis* (Orr et al., 2013; Tocheri et al., 2007) suggest that the carpus of hominins lacked modern human‐like carpus morphology until late in the hominin lineage, supporting the finding of the current study. More research examining and quantifying the carpal morphology of these fossil hominins in the context of anthropoid primate carpal morphology may shed further light on the timing and trajectory of hominin carpus evolution.

Despite the evidence that Euclidean distances can shed light on specialization, our method finds that the carpals of knuckle walkers are not morphologically distinct – or specialized—within the broader context of other anthropoids, often falling near the center of the PCA morphospace (e.g., Figure 8) and consistently possessing low Euclidean distances from the carpals of other locomotor groups both for individual carpals and the carpus as a whole (Figures 10 and 11; Table S8). Although this may appear in conflict with evidence that knuckle‐walking taxa may have a distinct combination of traits (e.g., a radially expanded capitate head and a waisted capitate neck), it is consistent with evidence that the carpals of knuckle‐walking taxa lack any specializations that cannot be found in any other group. These results in combination indicate that the individual carpals and full carpus of knuckle walkers are not particularly specialized (**Use Type 4**), a finding with implications for how we assess the likelihood of various evolutionary scenarios associated with the origin of the knuckle‐walking behavior in hominines.

If the carpals of knuckle walkers do not form a highly modified structure relative to those of other anthropoids and especially other hominoids, then comparatively fewer modifications to a hypothesized ancestral hominoid wrist may be necessary to evolve a wrist that facilitates knuckle walking as a behavior. As such, it may be relatively easy for knuckle‐walking behaviors to evolve independently in apes. Sufficiently strong selective pressure can cause several adaptations or specializations to evolve in parallel or convergently. Although outside the scope of this discussion, such a scenario has been hypothesized in the origins of hominoid orthogrady and/or below‐branch suspensory behaviors, which appear to require several morphological changes across the entire skeleton (Begun, 2007; Hunt, 2016). If early panins or gorillins evolved from arboreal specialists, the evolution of effective terrestrial behaviors would greatly expand their niches and grant escape from a “specialization trap” (Almécija et al., 2021). The benefit of an expanded niche combined with very little need for morphological change provides a compelling case that knuckle walking could easily evolve multiple times in apes. Similar arguments to this one have been made in support of hypothesized knuckle‐walking behaviors in the fossil hominid *Sivapithecus* (Begun & Kivell, 2011). Likewise, some functional/biomechanical arguments that have been made for how and why knuckle walking evolved in *Pan* and *Gorilla* (Lovejoy, Simpson, et al., 2009; Lovejoy, Suwa, et al., 2009; Simpson et al., 2018; Tuttle, 1969) can be interpreted as evidence that it evolved as a response to selective pressures to terrestrial quadrupedalism in taxa with existing adaptations for orthogrady, vertical climbing, or below‐branch behaviors such as arm hanging.

Simultaneously, however, a relatively unspecialized carpus in knuckle‐walking taxa suggests that African ape carpal morphology is certainly not too specialized to reasonably approximate the ancestral carpal morphology of the earliest hominins, whereas the carpus of modern humans is clearly specialized (**Use Type 4**). Particularly in the case of the capitate, scaphoid, and trapezoid, modern human carpals are generally more morphologically distinct—and thus, presumably, modified or specialized—than those of knuckle‐walking taxa (Figure 11; Table S8). These results are supported by fossil hominin capitates, scaphoid, and trapezoids described in the literature as African ape‐like and lacking later *Homo*‐like features such as a radioulnar wide capitate neck, an open capitate facet on the scaphoid, and radioulnar expansion of the trapezoid's palmar beak (Bush et al., 1982; Johanson, Lovejoy, et al., 1982; Kibii et al., 2011; Marzke, 1983; McHenry, 1983; Mongle et al., 2025; Orr et al., 2013; Ward et al., 1999). The traits that appear modified in knuckle walkers relative to other nonhuman anthropoids or hominoids are largely shared with humans and fossil hominins (Clark, 1947; Kivell et al., 2015; Kivell, Churchill, et al., 2018; Tocheri et al., 2007), and knuckle‐walking carpals are consistently the most similar morphologically to those of humans among all groups (Figure 10; Table S8). Although it is possible that phylogenic history rather than adaptive evolution drives this similarity, the fact remains that the carpals of knuckle walkers are both less derived or specialized than those of humans and have the lowest Euclidean distances to human carpals of any locomotor group. This presents strong evidence that the human carpus derived from an ancestor with a wrist morphology similar to that of the African apes, regardless of whether that morphology was specifically adapted to knuckle walking.

## 5 CONCLUSIONS

Here, we found that most purported knuckle‐walking traits of the carpus are not, in fact, reliable indicators of knuckle walking. Several of these features appear common across anthropoids or, instead, appear to be reliable indicators of suspensory behavior. If these features facilitate knuckle walking in African apes, they are likely *functional* but not *evolutionary* knuckle‐walking traits, primitive in African apes and recruited for this behavior. Although African apes appear to possess a unique combination of traits that characterize their carpal morphology (e.g., capitate head radial expansion with a narrow capitate neck), there is no single morphological feature which unites knuckle walkers to the exclusion to all other locomotor groups. The current study quantifiably observed that capitate head radial expansion is a hominine synapomorphy and that overall lunate morphology is strikingly similar in humans and African apes. One possible hypothesis to explain these hominine synapomorphies—and scaphoid–centrale fusion—is the knuckle‐walking screw‐clamp mechanism, although exploration of alternative hypotheses—such as vertical climbing (Almécija et al., 2021)—is warranted.

In addition to the purported knuckle‐walking traits shared between humans and African apes, we found that the carpals of knuckle walkers are not particularly morphologically distinct among anthropoid locomotor groups. In contrast to the capitate of bipedal humans, the scaphoid of digitigrade monkeys, or the hamate of brachiating hylobatids, no individual carpal of knuckle‐walking African apes is morphologically distinct (i.e., relationally specialized). Furthermore, African ape carpal bones evaluated as a whole appear unspecialized (Use Type 4) relative to those of bipeds, brachiators, and digitigrade quadrupeds, and modern human carpal bones are morphologically closest to those of African apes (Figures 10, 11). In conjunction, these results indicate that African ape carpal bones are not particularly specialized for knuckle walking and serve as the best approximation of ancestral hominin carpal bones among extant primates.

Our study provides the most comprehensive quantitative analysis of anthropoid carpal morphology and investigation of purported knuckle‐walking traits to date, and its ultimate finding is that there may, in fact, be no discernable definitive knuckle‐walking traits present in the carpus and thus there is no true “knuckle‐walking carpus.” This does not mean, however, that the carpus holds no clues for inferring the forelimb biomechanics of the panin–hominin LCA or any other fossil anthropoid. Additional work evaluating forelimb morphology in a phylogenetic context may help to elucidate (1) whether hominine synapomorphies evolved in response to selective pressures, and (2) the polarity of morphological similarities between African apes and monkeys to the exclusion of Asian apes (i.e., did Asian apes converge with each other or did African apes converge with monkeys?). Further studies of forelimb biomechanics focused on specific or compound movements and the relationships between individual bony elements (e.g., Orr et al., 2023) or across all elements may reveal competing hypotheses for the evolution of hominine wrist synapomorphies.

## AUTHOR CONTRIBUTIONS

L.E.H. and Z.A. conceived the study. L.E.H, M.W.T., B.A.P., and C.M.O. collected the sample. L.E.H. processed μCT and laser scan data, analyzed the data, performed statistical analyses, and drafted the manuscript. M.W.T., B.A.P., and C.M.O. contributed to processing uCT and laser scan data. All authors interpreted results and contributed to manuscript writing and editing.

## CONFLICT OF INTEREST STATEMENT

The authors have no conflict of interest to declare.

## Supporting information


**Figure S1:** Demonstration of scaphoid–centrale fusion of a *Pongo pygmaeus* specimen (top) and of prepollex removal in a *Gorilla beringei* specimen (bottom).
**Figure S2:** Landmarks applied to orient all scaphoids homologously for input into SPHARM analysis. Left: Description of each landmark's location. Right: Visualization of applied landmarks on a *Homo sapiens* scaphoid.
**Figure S3:** Landmarks applied to orient all lunates homologously for input into SPHARM analysis. Left: Description of each landmark's location. Right: Visualization of applied landmarks on a *Homo sapiens* lunate.
**Figure S4:** Landmarks applied to orient all triquetra homologously for input into SPHARM analysis. Left: Description of each landmark's location. Right: Visualization of applied landmarks on a *Homo sapiens* triquetrum.
**Figure S5:** Landmarks applied to orient all trapezia homologously for input into SPHARM analysis. Left: Description of each landmark's location. Right: Visualization of applied landmarks on a *Homo sapiens* trapezium.
**Figure S6:** Landmarks applied to orient all trapezoids homologously for input into SPHARM analysis. Left: Description of each landmark's location. Right: Visualization of applied landmarks on a *Homo sapiens* trapezoid.
**Figure S7:** Landmarks applied to orient all capitates homologously for input into SPHARM analysis. Left: Description of each landmark's location. Right: Visualization of applied landmarks on a *Homo sapiens* capitate.
**Figure S8:** Landmarks applied to orient all hamates homologously for input into SPHARM analysis. Left: Description of each landmark's location. Right: Visualization of applied landmarks on a *Homo sapiens* hamate.
**Figure S9:** Meshes corresponding to scaphoid morphology associated with extreme values along PC1 and PC2 (left) and individuals in the sample located at these extremes (right). Lower values of PC1 are associated with proximodistally short, dorsopalmarly deep scaphoids with long tubercles, whereas higher values of PC1 are associated with proximodistally long, dorsopalmarly deep scaphoids with short tubercles. Lower values of PC2 are associated with open capitates facets and absence of a ridge between the radius and trapeziotrapezoid facets, whereas higher values of PC2 are associated with a closed capitate facet and presence of a ridge between the radius and trapeziotrapezoid facets. All meshes are displayed in six anatomical views (from left to right): (1) palmar (tubercle), (2) radial (radial facet), (3) dorsal (radial facet), (4) ulnar (capitate and lunate facets), (5) proximal (radial facet), and (6) distal (trapezoidal and trapezial facets). Distal is up in palmar, radial, dorsal, and ulnar views. Palmar is up in proximal view, and dorsal is up in distal view.
**Figure S10:** Meshes corresponding to lunate morphology associated with extreme values along PC1 and PC2 (left) and individuals in the sample located at these extremes (right). Lower values of PC1 are associated with radioulnarly wide lunates with capitate facets in‐line with the lunate body, whereas higher values of PC1 are associated with radioulnarly narrow lunates with capitate facets that appear radially displaced relative to the lunate body. PC2 are associated with defined scaphoid facets and radioulnarly wide, proximoulnarly listing distal facets with more hamate than capitate articulation. whereas higher values of PC2 are associated with a lack of scaphoid facet definition and radioulnarly narrow distal facets with no hamatolunate contact. All meshes are displayed in six anatomical views (from left to right): (1) palmar, (2) radial (scaphoid facet), (3) dorsal, (4) ulnar (triquetral facet), (5) proximal (radial facet), and (6) distal (capitate and hamate facets). Distal is up in palmar, radial, dorsal, and ulnar views. Palmar is up in proximal view, and dorsal is up in distal view.
**Figure S11:** Meshes corresponding to triquetrum morphology associated with extreme values along PC1 and PC2 (left) and individuals in the sample located at these extremes (right). Lower values of PC1 are associated with dorsopalmarly deep triquetra with ulnar articulation and large, proximally oriented pisiform facets, whereas higher values of PC1 are associated with proximodistally long triquetra with small, palmarly oriented pisiform facets. Lower values of PC2 are associated with a pointed, nonarticular ulnodistal extension and convex lunate facets, whereas higher values of PC2 are associated with a lack of nonarticular ulnodistal protuberance and concave lunate facets. All meshes are displayed in six anatomical views (from left to right): (1) palmar (pisiform facet), (2) radial (hamate and lunate facets), (3) dorsal, (4) ulnar (ulnar facet), (5) proximal, and (6) distal (hamate facet). Distal is up in palmar, radial, dorsal, and ulnar views. Palmar is up in proximal view, and dorsal is up in distal view.
**Figure S12:** Meshes corresponding to trapezium morphology associated with extreme values along PC1 and PC2 (left) and individuals in the sample located at these extremes (right). Lower values of PC1 are associated with proximodistally short, dorsopalmarly deep trapezia with large tubercles, whereas higher values of PC1 are associated with proximodistally long, dorsopalmarly shallow trapezia with small tubercles. Lower values of PC2 are associated with large, palmarly expanded, saddle‐shaped first metacarpal, whereas higher values of PC2 are associated with radially convex first metacarpal facets. All meshes are displayed in six anatomical views (from left to right): (1) palmar (tubercle and metacarpal 1 facets), (2) radial (metacarpal 1 facet), (3) dorsal, (4) ulnar, (5) proximal (scaphoid and trapezoid facets), and (6) distal (metacarpal 1 facet). Distal is up in palmar, radial, dorsal, and ulnar views. Palmar is up in proximal view, and dorsal is up in distal view.
**Figure S13:** Meshes corresponding to trapezoid morphology associated with extreme values along PC1 and PC2 (left) and individuals in the sample located at these extremes (right). Lower values of PC1 are associated with dorsopalmarly deep trapezoids with more planar, distally oriented second metacarpal facets and radioulnarly broad palmar beaks, whereas higher values of PC1 are associated with dorsopalmarly shallow trapezoids with more mediolaterally oriented second metacarpal facets and extremely tapered palmar beaks resulting from acutely angled scaphoid and trapezium facets. Lower values of PC2 are associated with a scaphoid facet with a palmar extent that lists more palmoradially, whereas higher values of PC2 are associated with a scaphoid facet with a palmar extent that does not list palmoradially. All meshes are displayed in six anatomical views (from left to right): (1) palmar (trapezial and metacarpal 2 facets), (2) radial (trapezial facet), 3) dorsal, (4) ulnar, (5) proximal (scaphoid facet), and (6) distal (metacarpal 2 facets). Distal is up in palmar, radial, dorsal, and ulnar views. Palmar is up in proximal view, and dorsal is up in distal view.
**Figure S14:** Meshes corresponding to capitate morphology associated with extreme values along PC1 and PC2 (left) and individuals in the sample located at these extremes (right). PC1 is in palmar view with distal up. Lower values of PC1 are associated with radioulnarly wide capitate heads and necks, whereas higher values of PC1 are associated with radioulnarly narrow, “waisted” necks and heads. Lower values of PC2 are associated with dorsopalmarly flat but complex third metacarpal facets and palmarly restricted second metacarpal facets, whereas higher values of PC2 are associated with dorsopalmarly “cupped” but simple third metacarpal facets and second metacarpal facets spanning the entire dorsopalmar length of the bone. All meshes are displayed in six anatomical views (from left to right): (1) palmar, (2) radial (scaphoid and trapezoid facets), (3) dorsal (lunate facet), (4) ulnar (hamate facet), (5) proximal (capitate head), and (6) distal (metacarpal 3 facet). Distal is up in palmar, radial, dorsal, and ulnar views. Palmar is up in proximal view, and dorsal is up in distal view.
**Figure S15:** Meshes corresponding to hamate morphology associated with extreme values along PC1 and PC2 (left) and individuals in the sample located at these extremes (right). PC1 is in palmar view with distal up. Lower values of PC1 are associated with proximodistally long hamates with distally oriented hamuli and entirely convex triquetral facets, whereas higher values of PC1 are associated with radioulnarly wide hamates with palmarly oriented hamuli and concavoconvex triquetral facets. Lower values of PC2 are associated with large hamuli and dorsopalmarly flat metacarpal facets, whereas higher values of PC2 are associated with small hamuli and highly dorsopalmarly concave metacarpal facets. All meshes are displayed in six anatomical views (from left to right): (1) palmar (hamulus), (2) radial (capitate facet), (3) dorsal, (4) ulnar (triquetral facet), (5) proximal (capitate and triquetral facets), and (6) distal (metacarpals 4 and 5 facets). Distal is up in palmar, radial, dorsal, and ulnar views. Palmar is up in proximal view, and dorsal is up in distal view.
**Figure S16:** PCA phylomorphospace/scatterplot and box‐and‐whisker plots of PCs 3 and 4 for scaphoid, lunate, triquetrum, trapezium, trapezoid, capitate, and hamate. Morphs are presented in optimal orientations for viewing differences along each PC axis. See below descriptions for further details.
**Table S1:** Results (*p*‐values) of Wilcox tests with Bonferroni *p*‐adjustment evaluating differences/discrimination between locomotor behavior groups based on scaphoid morphology (PC1–PC20). Statistically significant differences (*α* = 0.05) highlighted in red. Vertical line delineates PCs which describe >5% from those which describe <5%.
**Table S2:** Results (*p*‐values) of Wilcox tests with Bonferroni *p*‐adjustment evaluating differences/discrimination between locomotor behavior groups based on lunate morphology (PC1–PC20). Statistically significant differences (*α* = 0.05) highlighted in red. Vertical line delineates PCs which describe >5% from those which describe <5%.
**Table S3:** Results (*p*‐values) of Wilcox tests with Bonferroni *p*‐adjustment evaluating differences/discrimination between locomotor behavior groups based on triquetrum morphology (PC1–PC20). Statistically significant differences (*α* = 0.05) highlighted in red. Vertical line delineates PCs which describe >5% from those which describe <5%.
**Table S4:** Results (*p*‐values) of Wilcox tests with Bonferroni *p*‐adjustment evaluating differences/discrimination between locomotor behavior groups based on trapezium morphology (PC1–PC20). Statistically significant differences (*α* = 0.05) highlighted in red. Vertical line delineates PCs which describe >5% from those which describe <5%.
**Table S5:** Results (*p*‐values) of Wilcox tests with Bonferroni *p*‐adjustment evaluating differences/discrimination between locomotor behavior groups based on trapezoid morphology (PC1–PC20). Statistically significant differences (*α* = 0.05) highlighted in red. Vertical line delineates PCs which describe >5% from those which describe <5%.
**Table S6:** Results (s) of Wilcox tests with Bonferroni p‐adjustment evaluating differences/discrimination between locomotor behavior groups based on capitate morphology (PC1–PC20). Statistically significant differences (*α* = 0.05) highlighted in red. Vertical line delineates PCs which describe >5% from those which describe <5%.
**Table S7:** Results (*p*‐values) of Wilcox tests with Bonferroni *p*‐adjustment evaluating differences/discrimination between locomotor behavior groups based on hamate morphology (PC1–PC20). Statistically significant differences (*α* = 0.05) highlighted in red. Vertical line delineates PCs which describe >5% from those which describe <5%.
**Figure S17:** Mean distance across all pairwise comparisons between locomotor behaviors (green) and magnitude of variance in mean distance between each pairwise comparisons (yellow). Green: lighter colors indicate that a carpal element is more morphologically distinct across locomotor behaviors. Yellow: lighter colors indicate that a carpal element is very similar between some locomotor behavior classes and very different between others.
**Table S8:** Mean Euclidean distance for each individual carpal element (blue) and for the entire carpus as a unit (red). Darker colors indicate greater similarity. Mean distance across all pairwise comparisons between locomotor behaviors (green) and magnitude of variance in mean distance between each pairwise comparisons (yellow). Green: lighter colors indicate that a carpal element is more morphologically distinct across locomotor behaviors. Yellow: lighter colors indicate that a carpal element is very similar between some locomotor behavior classes and very different between others.

## Data Availability

The data that support the findings of this study are available from the corresponding author upon reasonable request.
