# Supplementary material for: African ape carpal morphology is not too specialized to represent the ancestral hominin carpus
Source: J Anat. 2026 Sep 17:10.1111/joa.70241. Online ahead of print. doi: 10.1111/joa.70241 (PMC13586067; doi:10.1111/joa.70241)
Supplement: Supplementary file 1 [file JOA-9999-0-s001.docx]

**SUPPLEMENTARY MATERIAL**

**Figure S1:** Demonstration of scaphoid-centrale fusion of a *Pongo pygmaeus* specimen (top) and of prepollex removal in a *Gorilla beringei* specimen (bottom).

**Figure S2:** Landmarks applied to orient all scaphoids homologously for input into SPHARM analysis. **Left:** Description of each landmark’s location. **Right:** Visualization of applied landmarks on a *Homo sapiens* scaphoid.

**Figure S3:** Landmarks applied to orient all lunates homologously for input into SPHARM analysis. **Left:** Description of each landmark’s location. **Right:** Visualization of applied landmarks on a *Homo sapiens* lunate.

**Figure S4:** Landmarks applied to orient all triquetra homologously for input into SPHARM analysis. **Left:** Description of each landmark’s location. **Right:** Visualization of applied landmarks on a *Homo sapiens* triquetrum.

**Figure S5:** Landmarks applied to orient all trapezia homologously for input into SPHARM analysis. **Left:** Description of each landmark’s location. **Right:** Visualization of applied landmarks on a *Homo sapiens* trapezium.

**Figure S6:** Landmarks applied to orient all trapezoids homologously for input into SPHARM analysis. **Left:** Description of each landmark’s location. **Right:** Visualization of applied landmarks on a *Homo sapiens* trapezoid.

**Figure S7:** Landmarks applied to orient all capitates homologously for input into SPHARM analysis. **Left:** Description of each landmark’s location. **Right:** Visualization of applied landmarks on a *Homo sapiens* capitate.

**Figure S8:** Landmarks applied to orient all hamates homologously for input into SPHARM analysis. **Left:** Description of each landmark’s location. **Right:** Visualization of applied landmarks on a *Homo sapiens* hamate.

 **Figure S9:** Meshes corresponding to scaphoid morphology associated with extreme values along PC1 and PC2 (left) and individuals in the sample located at these extremes (right). Lower values of PC1 are associated with proximodistally short, dorsopalmarly deep scaphoids with long tubercles, whereas higher values of PC1 are associated with proximodistally long, dorsopalmarly deep scaphoids with short tubercles. Lower values of PC2 are associated with open capitates facets and absence of a ridge between the radius and trapeziotrapezoid facets, whereas higher values of PC2 are associated with a closed capitate facet and presence of a ridge between the radius and trapeziotrapezoid facets. All meshes are displayed in six anatomical views (from left to right): 1) palmar (tubercle), 2) radial (radial facet), 3) dorsal (radial facet), 4) ulnar (capitate and lunate facets), 5) proximal (radial facet), and 6) distal (trapezoidal and trapezial facets). Distal is up in palmar, radial, dorsal, and ulnar views. Palmar is up in proximal view, and dorsal is up in distal view.

**Figure S10:** Meshes corresponding to lunate morphology associated with extreme values along PC1 and PC2 (left) and individuals in the sample located at these extremes (right). Lower values of PC1 are associated with radioulnarly wide lunates with capitate facets in line with the lunate body, whereas higher values of PC1 are associated with radioulnarly narrow lunates with capitate facets that appear radially displaced relative to the lunate body. PC2 are associated with defined scaphoid facets and radioulnarly wide, proximoulnarly listing distal facets with more hamate than capitate articulation. whereas higher values of PC2 are associated with a lack of scaphoid facet definition and radioulnarly narrow distal facets with no hamatolunate contact. All meshes are displayed in six anatomical views (from left to right): 1) palmar, 2) radial (scaphoid facet), 3) dorsal, 4) ulnar (triquetral facet), 5) proximal (radial facet), and 6) distal (capitate and hamate facets). Distal is up in palmar, radial, dorsal, and ulnar views. Palmar is up in proximal view, and dorsal is up in distal view.

**Figure S11:** Meshes corresponding to triquetrum morphology associated with extreme values along PC1 and PC2 (left) and individuals in the sample located at these extremes (right). Lower values of PC1 are associated with dorsopalmarly deep triquetra with ulnar articulation and large, proximally oriented pisiform facets, whereas higher values of PC1 are associated with proximodistally long triquetra with small, palmarly oriented pisiform facets. Lower values of PC2 are associated with a pointed, nonarticular ulnodistal extension and convex lunate facets, whereas higher values of PC2 are associated with a lack of nonarticular ulnodistal protuberance and concave lunate facets. All meshes are displayed in six anatomical views (from left to right): 1) palmar (pisiform facet), 2) radial (hamate and lunate facets), 3) dorsal, 4) ulnar (ulnar facet), 5) proximal, and 6) distal (hamate facet). Distal is up in palmar, radial, dorsal, and ulnar views. Palmar is up in proximal view, and dorsal is up in distal view.

 **Figure S12:** Meshes corresponding to trapezium morphology associated with extreme values along PC1 and PC2 (left) and individuals in the sample located at these extremes (right). Lower values of PC1 are associated with proximodistally short, dorsopalmarly deep trapezia with large tubercles, whereas higher values of PC1 are associated with proximodistally long, dorsopalmarly shallow trapezia with small tubercles. Lower values of PC2 are associated with large, palmarly expanded, saddle shaped first metacarpal, whereas higher values of PC2 are associated with radially convex first metacarpal facets. All meshes are displayed in six anatomical views (from left to right): 1) palmar (tubercle and metacarpal 1 facets), 2) radial (metacarpal 1 facet), 3) dorsal, 4) ulnar, 5) proximal (scaphoid and trapezoid facets), and 6) distal (metacarpal 1 facet). Distal is up in palmar, radial, dorsal, and ulnar views. Palmar is up in proximal view, and dorsal is up in distal view.

**Figure S13:** Meshes corresponding to trapezoid morphology associated with extreme values along PC1 and PC2 (left) and individuals in the sample located at these extremes (right). Lower values of PC1 are associated with dorsopalmarly deep trapezoids with more planar, distally oriented second metacarpal facets and radioulnarly broad palmar beaks, whereas higher values of PC1 are associated with dorsopalmarly shallow trapezoids with more mediolaterally oriented second metacarpal facets and extremely tapered palmar beaks resulting from acutely angled scaphoid and trapezium facets. Lower values of PC2 are associated with a scaphoid facet with a palmar extent that lists more palmoradially, whereas higher values of PC2 are associated with a scaphoid facet with a palmar extent that does not list palmoradially. All meshes are displayed in six anatomical views (from left to right): 1) palmar (trapezial and metacarpal 2 facets), 2) radial (trapezial facet), 3) dorsal, 4) ulnar, 5) proximal (scaphoid facet), and 6) distal (metacarpal 2 facets). Distal is up in palmar, radial, dorsal, and ulnar views. Palmar is up in proximal view, and dorsal is up in distal view.

**Figure S14:** Meshes corresponding to capitate morphology associated with extreme values along PC1 and PC2 (left) and individuals in the sample located at these extremes (right). PC1 is in palmar view with distal up. Lower values of PC1 are associated with radioulnarly wide capitate heads and necks, whereas higher values of PC1 are associated with radioulnarly narrow, “waisted” necks and heads. Lower values of PC2 are associated with dorsopalmarly flat but complex third metacarpal facets and palmarly restricted second metacarpal facets, whereas higher values of PC2 are associated with dorsopalmarly “cupped” but simple third metacarpal facets and second metacarpal facets spanning the entire dorsopalmar length of the bone. All meshes are displayed in six anatomical views (from left to right): 1) palmar, 2) radial (scaphoid and trapezoid facets), 3) dorsal (lunate facet), 4) ulnar (hamate facet), 5) proximal (capitate head), and 6) distal (metacarpal 3 facet). Distal is up in palmar, radial, dorsal, and ulnar views. Palmar is up in proximal view, and dorsal is up in distal view.

**Figure S15:** Meshes corresponding to hamate morphology associated with extreme values along PC1 and PC2 (left) and individuals in the sample located at these extremes (right). PC1 is in palmar view with distal up. Lower values of PC1 are associated with proximodistally long hamates with distally oriented hamuli and entirely convex triquetral facets, whereas higher values of PC1 are associated with radioulnarly wide hamates with palmarly oriented hamuli and concavoconvex triquetral facets. Lower values of PC2 are associated with large hamuli and dorsopalmarly flat metacarpal facets, whereas higher values of PC2 are associated with small hamuli and highly dorsopalmarly concave metacarpal facets. All meshes are displayed in six anatomical views (from left to right): 1) palmar (hamulus), 2) radial (capitate facet), 3) dorsal, 4) ulnar (triquetral facet), 5) proximal (capitate and triquetral facets), and 6) distal (metacarpals 4 and 5 facets). Distal is up in palmar, radial, dorsal, and ulnar views. Palmar is up in proximal view, and dorsal is up in distal view.

**Figure S16:** PCA phylomorphospace/scatterplot and box-and-whisker plots of PCs 3 and 4 for scaphoid, lunate, triquetrum, trapezium, trapezoid, capitate, and hamate. Morphs are presented in optimal orientations for viewing differences along each PC axis. See below descriptions for further details.

**Description of PCs 3 and 4 depicted in Figure S16:**

Scaphoid: PC3 (Fig. S16: proximal view with palmar up) describes 10.9% of the variance in the sample and reflects the radioulnar width of the palmar scaphoid body where it meets the scaphoid tubercle (often called the scaphoid waist in clinical contexts). *Pan* species (negative PC3 scores) possess radioulnarly narrow scaphoid waists; all other taxa (positive PC3 scores) possess radioulnarly wide scaphoid waists. PC4 (Fig. S16 ulnar view with distal up) describes 5.5% of the variance in the sample and reflects the dorsopalmar curvature of the trapeziotrapezoid facet. *Pan* species and *Gorilla gorilla* (negative PC4 scores) possess dorsopalmarly curved trapeziotrapezoid facets; *Pongo* and palmigrade taxa (positive PC3 scores) possess dorsopalmarly flat trapeziotrapezoid facets.

Lunate: PC3 (Fig. S16: ulnar view with distal up) describes 8.1% of the variance in the sample and reflects the distal relative to proximal radioulnar breadth (i.e. “wedge” shape) and concavity of the triquetrum articular facet. *Pongo* species (negative PC3 scores) possess lunates which are radioulnarly wider proximally than they are distally and concave triquetrum articular facets; all other species (positive PC3 scores) possess more uniformly radioulnarly wide lunates with flat-to-convex triquetrum facets. PC4 (Fig. S16: proximal view with palmar up) describes 6.5% of the variance in the sample and reflects the size – particularly radioulnar breadth – of the facet for the radius and shows no clear taxonomic or functional trend.

Triquetrum: PC3 (Fig. S16: palmar view with distal up) describes 7.3% of the variance in the sample and reflects the 1) radioulnar breadth of the distal triquetrum as well as 2) the shape and proximal extent of the articular facet for the pisiform. *Pan* species (negative PC3 scores) possess triquetra with radioulnarly narrow distal ends and proximodistally long articular facets for the pisiform that extend down most of the proximodistal length of the palmar surface of the bone. *Homo sapiens* (positive PC3 scores) possess triquetra with radioulnarly wide distal ends and proximodistally short articular facets that terminate approximately halfway down the the proximodistal extent of the palmar surface of the bone. PC4 (Fig. S16: radiopalmar view with distal up) describes 6.0% of the variance in the sample and reflects the degree of concavity of the hamate and pisiform facets. Hylobatids (negative PC4 scores) possess more concave hamate and pisiform facets; *Pongo* species and *Ceropithecus mitis* (positive PC4 scores) possess relatively flat hamate and pisiform facets.

Trapezium: PC3 (Fig. S16: distal view with palmar up) describes 7.9% of the variance in the sample and reflects 1) the dorsopalmar curvature relative to the radioulnar curvature of the first metacarpal facet and 2) the “relief” of the first metacarpal facet (i.e., how contiguous the facet is with the surrounding bone). Knuckle walking and quadrumanuous taxa (negative PC3 scores) possess more dorsopalmarly curved first metacarpal facets that are in greater relief (i.e., not contiguous) from the surrounding bone; *Homo sapiens* and *Hoolock hoolock* (positive PC3 scores) possess less dorsopalmarly curved first metacarpal facets that are in less relief (i.e., contiguous) from the surrounding bone. In the trapezium, the palmar-most point of the articular facet for the first metacarpal often protrudes radiodistally relative to the surrounding bone. Concomitantly, the point at which the scaphoid and trapezoid articular facets meet forms a bony point that protrudes proximoulnarly. These two bony protrusions typically fall at approximately the same location along the dorsopalmar length of the trapezium, resulting in a “cross” or “t” shape. PC4 (Fig. S16: palmar view with distal up) describes 6.0% of the variance in the sample and is associated with the length and dorsopalmar position of these two bony protrusions. In palmigrade taxa (positive PC4 scores), these are longer and more dorsally placed than they are in other taxa (negative PC4 scores).

Trapezoid: PC3 (Fig. S16: radial view with distal up) describes 8.5% of the variance in the sample and reflects proximodistal length and dorsopalmar curvature of the articular facets for the second metacarpal. *Sapajus apella* (negative PC4 scores) possess proximodistally short trapezoids with dorsopalmarly flat articular facets for the second metacarpal; all other taxa (positive PC4 scores) possess proximodistally long trapezoids with dorsopalmarly concave articular facets for the second metacarpal. PC4 (Fig. S16: palmar view with distal up) describes 7.0% of the variance in the sample and reflects overall radioulnar breadth of the bone. Hylobatids and *Homo sapiens* (negative PC4 scores) possess trapezoids which are overall radioulnarly narrow; *Gorilla* taxa (positive PC4 scores) possess trapezoids which are overall radioulnarly wide.

Capitate: PC3 (Fig. S16: ulnar view with distal up) describes 10.0% of the variance in the sample and reflects 1) degree of projection of the palmodistal extent of the capitate and 2) overall “footprint” (i.e., dimensions) of articular facet for the third metacarpal. Hylobatids (negative PC3 scores) possess capitates with no projection of the palmodistal extent and radioulnarly wide articular facets for the third metacarpal; *Gorilla* species (positive PC3 scores) possess capitates with substantial projection of the palmodistal extent and radioulnarly narrow articular facets for the third metacarpal. PC4 (Fig. S16: dorsal view with distal up) describes 7.1% of the variance in the sample and reflects the distal extent of the dorsal side of the articular facet for the lunate. Hylobatids and *Gorilla* species (negative PC4 scores) possess articular facets of the lunate which terminate proximally on the dorsal surface of the capitate; *Pongo* species (positive PC4 scores) possess articular facets of the lunate which extend distally on the dorsal surface of the capitate.

Hamate: PC3 (Fig. S16: palmar view with distal up) describes 8.5% of the variance in the sample and reflects hamulus radioulnar width and depth of the fossa for the capitohamate ligament. *Homo sapiens* (negative PC3 scores) possess radioulnarly narrow hamuli and shallow fossae for the capitohamate ligament; *Gorilla* species (positive PC3 scores) possess radioulnarly wide hamuli and deep fossae for the capitohamate ligament. PC4 (Fig. S16: distal view with dorsal up) describes 5.0% of the variance in the sample distal height of the fourth metacarpal facet.

**Table S1: Results (p-values) of Wilcox tests with Bonferroni p-adjustment evaluating differences/discrimination between locomotor behavior groups based on scaphoid morphology (PC1-PC20). Statistically significant differences (α = 0.05) highlighted in red. Vertical line delineates PCs which describe >5% from those which describe <5%.**

**Table S2: Results (p-values) of Wilcox tests with Bonferroni p-adjustment evaluating differences/discrimination between locomotor behavior groups based on lunate morphology (PC1-PC20). Statistically significant differences (α = 0.05) highlighted in red. Vertical line delineates PCs which describe >5% from those which describe <5%.**

**Table S3: Results (p-values) of Wilcox tests with Bonferroni p-adjustment evaluating differences/discrimination between locomotor behavior groups based on triquetrum morphology (PC1-PC20). Statistically significant differences (α = 0.05) highlighted in red. Vertical line delineates PCs which describe >5% from those which describe <5%.**

**Table S4: Results (p-values) of Wilcox tests with Bonferroni p-adjustment evaluating differences/discrimination between locomotor behavior groups based on trapezium morphology (PC1-PC20). Statistically significant differences (α = 0.05) highlighted in red. Vertical line delineates PCs which describe >5% from those which describe <5%.**

**Table S5: Results (p-values) of Wilcox tests with Bonferroni p-adjustment evaluating differences/discrimination between locomotor behavior groups based on trapezoid morphology (PC1-PC20). Statistically significant differences (α = 0.05) highlighted in red. Vertical line delineates PCs which describe >5% from those which describe <5%.**

**Table S6: Results (p-values) of Wilcox tests with Bonferroni p-adjustment evaluating differences/discrimination between locomotor behavior groups based on capitate morphology (PC1-PC20). Statistically significant differences (α = 0.05) highlighted in red. Vertical line delineates PCs which describe >5% from those which describe <5%.**

**Table S7: Results (p-values) of Wilcox tests with Bonferroni p-adjustment evaluating differences/discrimination between locomotor behavior groups based on hamate morphology (PC1-PC20). Statistically significant differences (α = 0.05) highlighted in red. Vertical line delineates PCs which describe >5% from those which describe <5%.**

**Figure S17:** Mean distance across all pairwise comparisons between locomotor behaviors (green) and magnitude of variance in mean distance between each pairwise comparisons (yellow). Green: lighter colors indicate that a carpal element is more morphologically distinct across locomotor behaviors. Yellow: lighter colors indicate that a carpal element is very similar between some locomotor behavior classes and very different between others.

**Table S8: Mean Euclidean distance for each individual carpal element (blue) and for the entire carpus as a unit (red). Darker colors indicate greater similarity. Mean distance across all pairwise comparisons between locomotor behaviors (green) and magnitude of variance in mean distance between each pairwise comparisons (yellow). Green: lighter colors indicate that a carpal element is more morphologically distinct across locomotor behaviors. Yellow: lighter colors indicate that a carpal element is very similar between some locomotor behavior classes and very different between others.**
